# Synthesis, *in silico* and *in vitro* antimicrobial efficacy of substituted arylidene-based quinazolin-4(3*H*)-one motifs

**DOI:** 10.3389/fchem.2023.1264824

**Published:** 2023-09-25

**Authors:** Gbolahan O. Oduselu, Damilola V. Aderohunmu, Olayinka O. Ajani, Oluwadunni F. Elebiju, Temitope A. Ogunnupebi, Ezekiel Adebiyi

**Affiliations:** ^1^ Covenant University Bioinformatics Research (CUBRe), Covenant University, Ota, Ogun State, Nigeria; ^2^ Department of Chemistry, Covenant University, Ota, Ogun State, Nigeria; ^3^ Division of Applied Bioinformatics, German Cancer Research Center (DKFZ), Heidelberg, Germany

**Keywords:** ADMET, antibacterial activities, antifungal activities, bioactive analog, hydrazone, inhibition, molecular docking, molecular dynamics simulation

## Abstract

**Introduction:** Quinazolin-4(3*H*)-one derivatives have attracted considerable attention in the pharmacological profiling of therapeutic drug targets. The present article reveals the development of arylidene-based quinazolin-4(3*H*)-one motifs as potential antimicrobial drug candidates.

**Methods:** The synthetic pathway was initiated through thermal cyclization of acetic anhydride on anthranilic acid to produce 2-methyl-4H-3,1-benzoxazan-4-one 1, which (upon condensation with hydrazine hydrate) gave 3-amino-2-methylquinazolin-4(3*H*)-one 2. The reaction of intermediate 2 at its amino side arm with various benzaldehyde derivatives furnished the final products, in the form of substituted benzylidene-based quinazolin-4(3*H*)-one motifs 3a–l, and with thiophene-2-carbaldehyde to afford 3 m. The purified targeted products 3a–m were effectively characterized for structural authentication using physicochemical parameters, microanalytical data, and spectroscopic methods, including IR, UV, and ^1^H- and ^13^C-NMR, as well as mass spectral data. The substituted arylidene-based quinazolin-4(3*H*)-one motifs 3a–m were screened for both *in silico* and *in vitro* antimicrobial properties against selected bacteria and fungi. The in silico studies carried out consisted of predicted ADMET screening, molecular docking, and molecular dynamics (MD) simulation studies. Furthermore, *in vitro* experimental validation was performed using the agar diffusion method, and the standard antibacterial and antifungal drugs used were gentamicin and ketoconazole, respectively.

**Results and discussion:** Most of the compounds possessed good binding affinities according to the molecular docking studies, while MD simulation revealed their levels of structural stability in the protein–ligand complexes. 2-methyl-3-((thiophen-2-ylmethylene)amino) quinazolin-4(3*H*)-one 3 m emerged as both the most active antibacterial agent (with an minimum inhibitory concentration (MIC) value of 1.95 μg/mL) against Staphylococcus aureus and the most active antifungal agent (with an MIC value of 3.90 μg/mL) against *Candida albicans*, *Aspergillus niger*, and *Rhizopus nigricans*.

## 1 Introduction

Over the years, the natural occurrence of heterocyclic compounds in biological systems ranging from nucleic acids to protein has formed the basis for heterocyclic engineering and drug design (Kumar et al., 2016). The route to drug design formulation focuses on attempting to imitate heterocyclic compounds that are present in nature and explores their medicinal potential by deviating from the pathways established by nature (Ajani et al., 2017a; Berger et al., 2019). Quinazolin-4(3*H*)-one is an *N*-heterocyclic compound that belongs to the family of benzodiazines with its nitrogen heteroatom situated in the 1- and 3-positions and a carbonyl of cyclic amide in the 4-position (Rakesh et al., 2019). Researchers are motivated to incorporate diverse biologically active motifs into the quinazolinone system for novel drug design because of the high degree of stability of this template (Auti et al., 2020). Quinazolin-4(3*H*)-one is a heterocyclic motif of interest in therapeutic medicine owing to its broad range of reported pharmacological activities, which include antimicrobial (Rakesh et al., 2019), antimalarial (Chugh et al., 2020), anticancer (Li et al., 2023), antioxidant (Verma et al., 2022), antitumor (Ramadan et al., 2020), and histone deacetylases inhibitory (Dhuguru and Ghoneim, 2022) activities.

Furthermore, due to the broad horizon of material science applications and the wide spectrum of pharmacological potential exhibited by quinazolin-4(3*H*)-one, a vast number of synthetic approaches have been established to develop diverse arrays of quinazolinone motifs. These approaches include a mechanochemical method (Alam et al., 2018) and the thermal cyclization of 2-aminobenzamides and ortho-esters (Gavin et al., 2018). More recently, challenges associated with the ring opening of quinazolin-4(3*H*)-one have been overcome by an alternative fusion strategy (Patel et al., 2019). In our earlier review (Ajani et al., 2017a), it was noted that other valuable methods for achieving quinazolinone derivatives include the thermal cyclization of ureidobenzoic acid, ruthenium-catalyzed dehydrogenation, a cascade reaction of 2-(aminophenyl)methanol with aldehyde, a solid-phase-supported approach using cerium ammonium nitrate, a β-cyclodextrin-mediated MCR approach, and a microwave irradiation technique, among others.

The menacing issue of drug resistance, coupled with the relentless emergence of infectious diseases, has had a detrimental impact on global health (Gogoi et al., 2021). However, the advent of computational methods has contributed greatly to the prediction of the pharmacodynamics and pharmacokinetic properties of synthesized compounds even prior to pre-clinical and clinical trials (Acuna et al., 2020). Several computer-aided drug design (CADD) techniques (Lin et al., 2020), encompassing both ligand-based drug design (LBDD) and structure-based drug design (SBDD), are currently employed in the *in silico* prediction of the biological activities of compounds. Molecular docking is an approach within SBDD that is used to determine the binding conformations and affinities of small molecules with biological targets (Phillips et al., 2018), while molecular dynamics simulation is employed to evaluate the stability of molecules in the binding sites of biological targets (Oduselu et al., 2021a). These techniques are often used to support *in vitro* experimental testing of the compounds against target organisms (El-Adl et al., 2020). However, it is possible that a compound computationally predicted to be the best binder may not exhibit the best IC_50_ value upon experimental validation. Silva et al. (2017) have reported that, in many instances, 50%–70% of the best hits discovered via simple docking do not correlate well with those that perform effectively in *vitro* testing (Silva et al., 2017). As such, docking analyses are sometimes combined with further validation, such as molecular dynamics simulation (Phillips et al., 2018; Trust and Lilly, 2020).

Furthermore, there has been a rise in the incidence of bacterial and fungal infections in recent years (Zhao et al., 2021). The number of bacteria and fungi capable of causing severe illnesses in both humans and animals is extensive (Gnat et al., 2021). Although fatalities resulting from bacterial and fungal infections have decreased in developed countries, treating these infections in immunocompromised patients (e.g., patients with conditions such as COVID-19 or AIDs, those who have received organ transplants, or those who are undergoing anticancer therapy) remains a significant and intricate challenge for modern chemotherapy (Sharma et al., 2017; Rawson et al., 2021). Hence, it is both necessary and valuable to design novel quinazolin-4(3*H*)-one motifs bearing substituted benzylidene and thiophenylidene side moieties in search of bioactive motifs for the development of future antimicrobial (antibacterial and antifungal) drugs. This study also correlates the antimicrobial activities of the synthesized quinazolin-4(3*H*)-one derivatives based on their binding affinities with *in vitro* screening results.

## 2 Materials and methods

### 2.1 Chemistry

The reagents and chemicals used in this study were purchased from Sigma Aldrich (Germany). The progress of the reactions, purity level of the synthesized compounds, and examination of the mixture, when necessary, were monitored using thin-layer chromatography (TLC) spotting. The spots were visualized under a UV lamp and/or in an iodine crystal chamber. The melting points of all the solid products were determined using Stuart equipment. NMR spectra were recorded using both Bruker 400 MHz and 500 MHz spectrometers at room temperature, with chemical shifts (ppm) referenced against the internal standard, tetramethylsilane (TMS). Deuterated DMSO-d_6_ was used as the solvent, while ^1^H (400 MHz and 500 MHz), ^13^C (101MHz and 125 MHz), and 2D NMR (DEPT-135) spectroscopy were employed for structural elucidation of the synthesized compounds. FTIR spectroscopy analysis was conducted using a KBr (potassium bromide) pellet on a Perkin Elmer FT-IR Spectrophotometer, with the frequencies of absorption measured in wavenumber (cm^-1^), ranging from 4,000 cm^-1^ to 400 cm^-1^. Mass spectra were obtained using a Finnigan spectrometer operating in the EI mode at 70 eV.

#### 2.1.1 Synthesis of 2-methyl-4H-3,1-benzoxazin-4-one as precursor 1

Ethanol (EtOH) solvent (30 mL) was employed in the reaction between anthranilic acid (30 g, 220 mmol) and acetic anhydride. Anthranilic acid was stirred at room temperature to ensure complete dissolution. Acetic anhydride (30 mL) was added while stirring continuously. Due to noticeable heat release, the mixture was cooled on ice for a few minutes. The reaction was refluxed for 6 h, and its progress was monitored using TLC with dichloromethane (DCM) as the eluent. The resulting solution was filtered by suction and then dried to produce 2-methyl-4*H*-3,1-benzoxazin-4-one **1** as a gray-colored solid (95%, melting point = 171–173°C). ^1^H-NMR (500 MHz, DMSO-d_6_) δ_H_: 7.98–7.93 (d, *J* = 9.68 Hz, 1H of Ar-H), 7.76–7.72 (t, *J* = 9.68 Hz, 1H, Ar-H), 7.44–7.41 (t, *J* = 10.00 Hz, 1H, Ar-H), 7.43–7.39 (d, *J* = 10.00 Hz, 1H of Ar-H), 2.59 (s, 3H, CH_3_). ^13^C-NMR (125 MHz, DMSO-d_6_) δ_C_: 168.8 (C=O), 140.4 (O-C=N), 135.0, 130.7, 129.0 (CH), 127.8 (CH), 117.2 (CH), 114.5 (CH), 20.9 (CH_3_) ppm. DEPT 135 (125 MHz, DMSO-d_6_) δ_C_: +ve signals: 129.1 (CH), 127.9 (CH), 117.1 (CH), 114.0 (CH), 20.9 (CH_3_) ppm. -ve signals: nil.

#### 2.1.2 Synthesis of 3-amino-2-methylquinazolin-4(3*H*)-one as intermediate 2

A solution of precursor **1** (3 g, 18.63 mmol) in 10 mL of ethanol was stirred for few minutes, after which hydrazine hydrate (0.91 mL, 18.63 mmol) was cautiously added and the resulting solution was refluxed for 7 h (completion was monitored by TLC). The reaction mixture was cooled, filtered by suction, and air-dried to afford 3-amino-2-methylquinazolin-4(3*H*)-one **2**) as a gray-colored solid (98%, melting point = 224–226°C). ^1^H-NMR (500 MHz, DMSO-d_6_) δ_H_: 7.91–7.90 (d, *J* = 9.58 Hz, 2H), 7.30–7.27 (m, 2H), 6.50 (s, 2H, NH_2_), 2.07 (s, 3H, CH_3_). ^13^C-NMR (125 MHz, DMSO-d_6_) δ_C_: 173.2 (C=O), 151.2 (N-C=N), 135.0, 132.3, 122.9 (CH), 117.0 (CH), 116.8 (CH), 110.0 (CH), 20.8 (CH_3_) ppm. λ_max_ (mm)/log ε_max_ (mol^-1^ cm^-1^): 230 (5.443), 245 (5.439), 320 (5.089).

#### 2.1.3 General procedure for 3-(substituted-benzylideneamino)-2-methylquinazolin-4(3*H*)-one 3a–m

Intermediate **2** (0.50 g, 2.87 mmol) was dissolved in 15 mL of ethanol, and benzaldehyde derivatives (2.87 mmol) were added to a 250-mL round-bottom flask. The mixture was stirred at room temperature using a magnetic stirrer and then refluxed for the required period of time based on information from the TLC. The resulting mixtures were concentrated using a rotary evaporator. The obtained solid was purified via recrystallization or column chromatography, when necessary, yielding compounds **3a–m** in various colors.

##### 2.1.3.1 Synthesis of 3-(benzylideneamino)-2-methylquinazolin-4(3*H*)-one 3a

Reaction of intermediate **2** (0.50 g, 2.87 mmol) with benzaldehyde (0.29 mL, 2.87 mmol) gave brown-colored solid **3a** (0.71 g, 75%, melting point = 285 °C s)). ^1^H-NMR (400 MHz, DMSO-d_6_) δ_H_: 9.72 (s, 1H, N=C-H), 8.52–8.50 (d, *J* = 6.82 Hz, 1H, Aryl-H), 7.99–7.97 (d, *J* = 6.48 Hz, 1H, Aryl-H), 7.75–7.72 (d, *J* = 8.40 Hz, 2H, Aryl-H), 7.56–7.51 (m, 1H, Aryl-H), 7.12–7.08 (m, 1H, Aryl-H), 6.76–6.67 (m, 3H, Aryl-H), 2.12 (s, 3H, CH_3_) ppm. ^13^C-NMR (100 MHz, DMSO-d_6_) δ_C_: 172.7 (C=O), 157.0 (N-C=N), 154.4 (N=C-H), 150.8, 145.8, 141.7, 134.3 (CH), 131.3 (CH), 128.1 (2 × CH), 124.1, 122.2 (CH), 119.6 (CH), 116.0 (CH), 110.7 (CH), 22.5 (CH_3_) ppm. DEPT 135 (100 MHz, DMSO-d_6_) δ_C_: +ve signals: 154.4 (N=C-H), 134.3 (CH), 131.3 (CH), 128.1 (2 × CH), 122.2 (CH), 119.6 (CH), 116.0 (CH), 110.7 (CH), 22.5 (CH_3_) ppm. -ve signals: nil. FT-IR (KBr, cm^-1^): 3,020 (C-H aromatic), 2,924 (C-H aliphatic), 1,676 (C=O), 1,601 (C=C), 1,581 (C=N), 1,460, 1,376 (CH_3_ deformation), 1,282 (C-N), 980 (=C-H), 869 (C=C), 747 (Ar-H), 692. MS: in m/z [rel. %]: 283.12 [M—3H + Na, 65.5%], 263.11 [M, 75.0%], 262.13 [M—1, 30.1%], 249.12 [M—CH_2_, 77.5%], 248.11 [M—CH_3_, 50.5%], 160.05 [M—PhCN, 100.0%], 146.08 [M—PhCN—CH_2_, 90.2%], 15.04 [CH_3_^+^, 27.1%].

##### 2.1.3.2 Synthesis of 2-methyl-3-((2-nitrobenzylidene)amino)quinazolin-4(3*H*)-one 3b

Reaction of intermediate 2 (0.50 g, 2.87 mmol) with 2-nitrobenzaldehyde (0.43 g, 2.87 mmol) gave yellow-colored solid 3b (0.81 g, 91%, melting point = 157–159°C). ^1^H-NMR (400 MHz, DMSO-d_6_) δ_H_: 9.69 (s, 1H, N=C-H), 8.70–8.68 (d, *J* = 6.38 Hz, 1H, Aryl-H), 8.25–8.24 (d, *J* = 6.36 Hz, 1H, Aryl-H), 7.73–7.71 (d, *J* = 8.56 Hz, 1H, Aryl-H), 7.46–7.44 (d, *J* = 5.80 Hz, 1H, Aryl-H), 7.31–7.25 (m, 4H, Aryl-H). 2.10 (s, 3H, CH_3_). ^13^C-NMR (100 MHz, DMSO-d_6_) δ_C_: 172.1 (C=O), 160.4 (N-C=N), 155.2, 152.3 (N=C-H), 151.2, 147.0, 141.9 (CH), 138.7 (CH), 135.2 (CH), 132.3 (CH), 130.5 (CH), 127.3, 122.0 (CH), 116.7 (CH), 116.4 (CH), 20.8 (CH_3_) ppm. DEPT 135 (100 MHz, DMSO-d_6_) δ_C_: +ve signals: 152.3 (N=C-H), 141.9 (CH), 138.7 (CH), 135.2 (CH), 132.3 (CH), 130.5 (CH), 122.0 (CH), 116.7 (CH), 116.4 (CH), 20.8 (CH_3_) ppm. -ve signals: nil. UV-Vis.: λ_max_ (nm)/log ε_max_ (M^-1^ cm^-1^): 210 (4.32), 230 (4.41), 237 (4.38), 250 (4.41), 257 (5.11). IR (KBr, cm^-1^) ῡ: 3,079 (C-H aromatic), 2,912 (C-H aliphatic), 1,698 (C=O), 1,607 (C=C aromatic), 1,571 (C=N), 1,527 (N-O of NO_2_), 1,398 (CH_3_ deformation), 1,365 (NO_2_ symmetrical), 1,271 (C-N), 982 (=C-H bending), 743.

##### 2.1.3.3 Synthesis of 3-((2-chlorobenzylidene)amino)-2-methylquinazolin-4(3*H*)-one 3c

Reaction of intermediate 2 (0.50 g, 2.87 mmol) with 2-chloro benzaldehyde (0.32 mL, 2.87 mmol) gave cream-colored solid 3c (0.54 g, 64%, melting point = 130–132°C). ^1^H-NMR (400 MHz, DMSO-d_6_) δ_H_: 9.54 (s, 1H, N=C-H), 8.52–8.51 (d, *J* = 6.60 Hz, 1H, Aryl-H), 8.24–8.23 (d, *J* = 4.40 Hz, 1H, Aryl-H), 7.92–7.90 (d, *J* = 6.36 Hz, 1H, Aryl-H), 7.45–7.43 (d, *J* = 8.56 Hz, 1H, Aryl-H), 7.35–7.27 (m, 4H, Aryl-H), 2.07 (s, 3H, CH_3_). ^13^C-NMR (100 MHz, DMSO-d_6_) δ_C_: 172.3 (C=O), 158.8 (N-C=N), 153.2, 151.2, 149.7 (N=C-H), 144.7, 142.1 (CH), 136.6 (CH), 134.9 (CH), 132.3 (CH), 130.5 (CH), 127.0, 122.8 (CH), 122.0 (CH), 114.7 (CH), 20.7 (CH_3_) ppm. DEPT 135 (100 MHz, DMSO-d_6_) δ_C_: +ve signals: 149.7 (N=C-H), 142.1 (CH), 136.6 (CH), 134.9 (CH), 132.3 (CH), 130.5 (CH), 122.8 (CH), 122.0 (CH), 114.7 (CH), 20.7 (CH_3_) ppm. -ve signals: nil. UV-Vis.: λ_max_ (nm)/log ε_max_ (M^-1^ cm^-1^): 205 (3.80), 220 (1.23), 284 (4.05), 380 (3.76). IR (KBr, cm^-1^) ῡ: 3,113 (C-H of aromatic), 2,924 (C-H of aliphatic), 2,854 (C-H aliphatic), 1722 (C=O of ketone), 1701 (C=O of hydrazide), 1,649 (C=N hydrazone), 1,617 (C=C of aromatic), 1,231 (C-N), 783 (C-Cl).

##### 2.1.3.4 Synthesis of 3-((3-methoxybenzylidene)amino)-2-methylquinazolin-4(3*H*)-one 3d

Reaction of intermediate 2 (0.50 g, 2.87 mmol) with 3-methoxybenzaldehyde (0.35 mL, 2.87 mmol) gave cream-colored solid 3d (0.44 g, 52%, melting point = 237–239°C). ^1^H-NMR (400 MHz, DMSO-d_6_) δ_H_: 9.65 (s, 1H, N=C-H), 8.80 (s, 1H, Aryl-H), 8.48–8.46 (d, *J* = 8.32 Hz, 1H, Aryl-H), 8.22–8.20 (d, *J* = 6.36 Hz, 1H, Aryl-H), 7.98–7.96 (d, *J* = 7.84 Hz, 1H, Aryl-H), 7.86–7.84 (d, *J* = 6.36 Hz, 1H, Aryl-H), 7.57–7.54 (dd, *J*_1_ = 6.36 Hz, *J*_2_ = 8.32 Hz, 1H, Aryl-H), 7.14–7.10 (m, 2H, Aryl-H), 6.75–6.73 (d, *J* = 6.48 Hz, 1H, Aryl-H), 3.43 (s, 3H, OCH_3_), 2.11 (s, 3H, CH_3_). ^13^C-NMR (100 MHz, DMSO-d_6_) δ_C_: 170.8 (C=O), 157.0 (N-C=N), 154.2, 150.0 (N=C-H), 146.8, 141.5 (CH), 133.4 (CH), 130.4 (CH), 125.6, 124.3, 123.7 (CH), 120.7 (CH), 119.3 (CH), 116.9 (CH), 111.2 (CH), 44.5 (OCH_3_), 22.7 (CH_3_) ppm. DEPT 135 (100 MHz, DMSO-d_6_) δ_C_: +ve signals: 150.0 (N=C-H), 141.5 (CH), 133.4 (CH), 130.4 (CH), 123.7 (CH), 120.7 (CH), 119.3 (CH), 116.9 (CH), 111.2 (CH), 44.5 (OCH_3_), 22.7 (CH_3_) ppm. -ve signals: nil. IR (KBr, cm^-1^) ῡ: 3,028 (C-H of aromatic), 2,923 (C-H of aliphatic), 2,854 (C-H aliphatic), 1,697 (C=O), 1,617 (C=C of aromatic), 1,599 (C=N), 1,373 (CH_3_ deformation), 1,340 (O-CH_3_), 1,231 (C-N), 996 (=C-H), 726 (Ar-H).

##### 2.1.3.5 Synthesis of 3-((3-hydroxybenzylidene)amino)-2-methylquinazolin-4(3*H*)-one 3e

Reaction of intermediate 2 (0.50 g, 2.87 mmol) with 3-hydroxy benzaldehyde (0.35 g, 2.87 mmol) gave cream-colored solid 3e (0.80 g, 99%, melting point = 136–137°C). ^1^H-NMR (400 MHz, DMSO-d_6_) δ_H_: 9.85 (s, 1H, N=C-H), 8.89 (s, 1H, Aryl-H), 8.48–8.46 (d, *J* = 8.32 Hz, 1H, Aryl-H), 7.98–7.96 (d, *J* = 7.84 Hz, 1H, Aryl-H), 7.84–7.82 (d, *J* = 8.08 Hz, 1H, Aryl-H), 7.57–7.53 (dd, *J*_1_ = 8.08 Hz, *J*_2_ = 8.32 Hz, 1H, Aryl-H), 7.13–7.09 (m, 2H, Aryl-H), 6.76–6.74 (d, *J* = 8.96 Hz, 1H, Aryl-H), 6.52 (s, 1H, OH), 2.13 (s, 3H, CH_3_). ^13^C-NMR (100 MHz, DMSO-d_6_) δ_C_: 171.1 (C=O), 159.3 (N-C=N), 154.2 (C-O), 152.7 (N=C-H), 147.9, 139.9 (CH), 133.4 (CH), 129.6 (CH), 125.7, 124.9, 123.0 (CH), 121.1 (CH), 119.3 (CH), 115.2 (CH), 112.1 (CH), 22.5 (CH_3_) ppm. DEPT 135 (100 MHz, DMSO-d_6_) δ_C_: +ve signals: 152.7 (N=C-H), 139.9 (CH), 133.4 (CH), 129.6 (CH), 123.0 (CH), 121.1 (CH), 119.3 (CH), 115.2 (CH), 112.1 (CH), 22.5 (CH_3_) ppm. -ve signals: nil. UV-Vis.: λ_max_ (nm)/log ε_max_ (M^-1^ cm^-1^): 221 (4.45), 240 (4.59), 257 (4.98), 308 (4.91). IR (KBr, cm^-1^) ῡ: 3,459 (O-H), 3,106 (C-H aromatic), 2,923 (C-H aliphatic), 2,853 (C-H aliphatic), 1,699 (C=O), 1,622 (C=C aromatic), 1,580 (C=N), 1,393 (CH_3_ deformation), 1,346 (C-O), 1,245 (C-N), 927 (=C-H bending), 718 (Ar-H).

##### 2.1.3.6 Synthesis of 3-((4-chlorobenzylidene)amino)-2-methylquinazolin-4(3*H*)-one 3f

Reaction of intermediate 2 (0.50 g, 2.87 mmol) with 4-chlorobenzaldehyde (0.35 g, 2.87 mmol) gave brown-colored solid 3f (0.71 g, 84%, melting point = 162–163°C). ^1^H-NMR (400 MHz, DMSO-d_6_) δ_H_: 9.74 (s, 1H, N=C-H), 8.49–8.47 (d, *J* = 9.52 Hz, 2H, Aryl-H), 7.98–7.96 (d, *J* = 7.34 Hz, 1H, Aryl-H), 7.56–7.54 (d, *J* = 7.20 Hz, 1H, Aryl-H), 7.22–7.20 (d, *J* = 9.52 Hz, 2H, Aryl-H), 6.97–6.94 (m, 2H, Aryl-H), 2.13 (s, 3H, CH_3_). ^13^C-NMR (100 MHz, DMSO-d_6_) δ_C_: 171.8 (C=O), 158.7 (N-C=N), 155.7 (N=C-H), 153.8, 147.6, 140.2, 133.0 (2 × CH), 130.9, 128.4 (CH), 124.9 (CH), 116.9 (2 × CH), 113.8 (CH), 111.5 (CH), 22.3 (CH_3_) ppm. DEPT 135 (100 MHz, DMSO-d_6_) δ_C_: +ve signals: 155.7 (N=C-H), 133.0 (2 × CH), 128.4 (CH), 124.9 (CH), 116.9 (2 × CH), 113.8 (CH), 111.5 (CH), 22.3 (CH_3_) ppm. -ve signals: nil. UV-Vis.: λ_max_ (nm)/log ε_max_ (M^-1^ cm^-1^): 221 (4.35), 228 (4.26), 250 (4.45), 260 (4.81), 308 (4.76). IR (KBr, cm^-1^) ῡ: 3,001 (C-H aromatic), 2,942 (C-H aliphatic), 2,884 (C-H aliphatic), 1,689 (C=O), 1,616 (C=C aromatic), 1,571 (C=N), 1,334 (CH_3_ deformation), 1,275 (C-N), 935 (=C-H bending), 745 (Ar-H), 618 (C-Cl).

##### 2.1.3.7 Synthesis of 3-((4-(dimethylamino)benzylidene)amino)-2-methylquinazolin-4(3*H*)-one 3g

Reaction of intermediate 2 (0.50 g, 2.87 mmol) with 4-dimethyl aminobenzaldehyde (0.43 g, 2.87 mmol) gave orange-colored solid 3g (0.84 g, 95%, melting point = 116–117°C). ^1^H-NMR (400 MHz, DMSO-d_6_) δ_H_: 9.67 (s, 1H, N=C-H), 8.50–8.48 (d, *J* = 7.56 Hz, 1H, Aryl-H), 7.99–7.97 (d, *J* = 7.36 Hz, 1H, Aryl-H), 7.68–7.66 (d, *J* = 8.16 Hz, 2H, Aryl-H), 7.57–7.54 (dd, *J*_1_ = 7.56 Hz, *J*_2_ = 14.40 Hz, 1H, Aryl-H), 7.13–7.10 (dd, *J*_1_ = 7.36 Hz, *J*_2_ = 14.40 Hz, 1H, Aryl-H), 6.75–6.73 (d, *J* = 8.16 Hz, 2H, Aryl-H), 3.01 (s, 6H, 2 × CH_3_, -N(CH_3_)_2_), 2.13 (s, 3H, CH_3_). ^13^C-NMR (100 MHz, DMSO-d_6_) δ_C_: 172.7 (C=O), 159.7 (N-C=N), 156.6 (N=C-H), 154.4, 147.2, 142.1, 135.6 (2 × CH), 132.4, 128.1 (CH), 123.1 (CH), 119.3 (2 × CH), 116.2 (CH), 111.4 (CH), 40.8 (N(CH_3_)_2_), 22.5 (CH_3_) ppm. DEPT 135 (100 MHz, DMSO-d_6_) δ_C_: +ve signals: 156.6 (N=C-H), 135.6 (2 × CH), 128.1 (CH), 123.1 (CH), 119.3 (2 × CH), 116.2 (CH), 111.4 (CH), 40.8 (N(CH_3_)_2_), 22.5 (CH_3_) ppm. -ve signal: nil.

##### 2.1.3.8 Synthesis of 3-((4-hydroxybenzylidene)amino)-2-methylquinazolin-4(3*H*)-one 3 h

Reaction of intermediate 2 (0.50 g, 2.87 mmol) with 4-hydroxy benzaldehyde (0.35 g, 2.87 mmol) gave cream-colored solid 3 h (0.73 g, 91%, melting point = 263–265°C). ^1^H-NMR (400 MHz, DMSO-d_6_) δ_H_: 9.76 (s, 1H, N=C-H), 8.50–8.48 (d, *J* = 7.92 Hz, 1H, Aryl-H), 7.98–7.96 (d, *J* = 7.32 Hz, 1H, Aryl-H), 7.75–7.73 (d, *J* = 7.60 Hz, 2H, Aryl-H), 7.53–7.41 (m, 1H, Aryl-H), 7.16–7.09 (m, 1H, Aryl-H), 6.94–6.92 (d, *J* = 7.60 Hz, 2H, Aryl-H), 6.48 (s, 1H, OH), 2.12 (s, 3H, CH_3_). ^13^C-NMR (100 MHz, DMSO-d_6_) δ_C_: 172.9 (C=O), 159.3 (N-C=N), 157.2 (N=C-H), 155.1, 147.2, 142.1, 135.7 (2 × CH), 132.8, 128.1 (CH), 122.4 (CH), 119.0 (2 × CH), 115.8 (CH), 111.6 (CH), 22.5 (CH_3_) ppm. DEPT 135 (100 MHz, DMSO-d_6_) δ_C_: +ve signals are: 157.2 (N=C-H), 135.7 (2 × CH), 128.1 (CH), 122.4 (CH), 119.0 (2 × CH), 115.8 (CH), 111.6 (CH), 22.5 (CH_3_) ppm. +ve signals: nil. FT-IR (KBr, cm^-1^): 3,367 (OH), 3,394 (N-H), 3,020 (C-H aromatic), 2,924 (C-H aliphatic), 1,676 (C=O), 1,601 (C=C), 1,581 (C=N), 1,460, 1,376 (CH_3_ deformation), 1,282 (C-N), 980 (=C-H), 869 (C=C, out-of-plane bending), 747 (Ar-H, bending and ring puckering), 692.

##### 2.1.3.9 Synthesis of 3-((4-ethylbenzylidene)amino)-2-methylquinazolin-4(3*H*)-one 3i

Reaction of intermediate 2 (0.50 g, 2.87 mmol) with 4-ethylbenzaldehyde (0.38 mL, 2.87 mmol) gave brown-colored solid 3i (0.71 g, 84%, melting point = 160–162°C). ^1^H-NMR (400 MHz, DMSO-d_6_) δ_H_: 9.65 (s, 1H, N=C-H), 8.50–8.49 (d, *J* = 7.56 Hz, 1H, Aryl-H), 7.97–7.95 (d, *J* = 8.00 Hz, 1H, Aryl-H), 7.68–7.66 (d, *J* = 8.16 Hz, 2H, Aryl-H), 7.57–7.54 (m, 1H, Aryl-H), 7.13–7.10 (m, 1H, Aryl-H), 6.75–6.73 (d, *J* = 8.16 Hz, 2H, Aryl-H), 3.00–2.93 (q, *J* = 6.40 Hz, 2H, CH_2_CH_3_), 2.13 (s, 3H, CH_3_), 1.08–1.05 (t, *J* = 6.40 Hz, 3H, CH_3_CH_2_). ^13^C-NMR (100 MHz, DMSO-d_6_) δ_C_: 170.1 (C=O), 157.2 (N-C=N),151.9 (N=C-H), 141.4, 134.4, 132.5 (CH), 131.5 (CH), 128.8, 125.5 (2 × CH), 122.8 (CH), 120.2 (CH), 116.3 (2 × CH), 115.0, 33.4 (CH_2_), 25.4 (CH_3_), 13.9 (CH_3_) ppm. DEPT 135 (100 MHz, DMSO-d_6_) δ_C_: +ve signals: 151.9 (N=C-H), 132.5 (CH), 131.5 (CH), 125.5 (2 × CH), 122.8 (CH), 120.2 (CH), 116.3 (2 × CH), 25.4 (CH_3_), 13.9 (CH_3_) ppm. -ve signal: 33.4 (CH_2_) ppm.

##### 2.1.3.10 Synthesis of 3-((4-methoxybenzylidene)amino)-2-methylquinazolin-4(3*H*)-one 3j

Reaction of intermediate 2 (0.50 g, 2.87 mmol) with anisaldehyde (0.43 g, 2.87 mmol) gave brown-colored solid 3j (0.39 g, 46%, melting point = 275–277°C). ^1^H-NMR (400 MHz, DMSO-d_6_) δ_H_: 9.85 (s, 1H, N=C-H), 8.48–8.46 (d, *J* = 7.56 Hz, 1H, Aryl-H), 7.98–7.96 (d, *J* = 7.84 Hz, 1H, Aryl-H), 7.86–7.84 (d, *J* = 9.20 Hz, 2H, Aryl-H), 7.57–7.53 (m, 1H, Aryl-H), 7.22–7.20 (d, *J* = 9.20 Hz, 2H, Aryl-H), 7.14–7.10 (m, 1H, Aryl-H), 3.84 (s, 3H, O-CH_3_), 2.13 (s, 3H, CH_3_). ^13^C-NMR (100 MHz, DMSO-d_6_) δ_C_: 169.2 (C=O), 155.1 (N-C=N), 154.6 (N=C-H), 133.4, 132.0 (2 × CH), 130.4, 125.7, 124.9, 122.6 (CH), 119.3 (CH), 113.8 (2 × CH), 113.0 (CH), 111.5 (CH), 40.1 (OCH_3_), 22.4 (CH_3_) ppm. DEPT 135 (100 MHz, DMSO-d_6_) δ_C_: +ve signals: 154.6 (N=C-H), 132.0 (2 × CH), 122.6 (CH), 119.3 (CH), 113.8 (2 × CH), 113.0 (CH), 111.5 (CH), 40.1 (OCH_3_), 22.4 (CH_3_) ppm. -ve signals: nil.

##### 2.1.3.11 Synthesis of 3-((4-ethoxybenzylidene)amino)-2-methylquinazolin-4(3*H*)-one 3 k

Reaction of intermediate **2** (0.50 g, 2.87 mmol) with 4-ethoxy benzaldehyde (0.43 g, 2.87 mmol) gave cream-colored solid 3k (0.41 g, 46%, melting point = 129–130°C). ^1^H-NMR (400 MHz, DMSO-d_6_) δ_H_: 8.44 (s, 1H, N=C-H), 8.20–8.18 (d, *J* = 8.00 Hz, 2H, Aryl-H), 7.78–7.77 (d, *J* = 5.64 Hz, 1H, Aryl-H), 7.56–7.55 (d, *J* = 5.76 Hz, 1H, Aryl-H), 7.23–7.21 (d, *J* = 7.92 Hz, 2H, Aryl-H), 7.10–7.07 (m, 2H, Aryl-H), 4.40–4.35 (q, *J* = 7.12 Hz, 2H, CH_2_CH_3_), 2.13 (s, 3H, CH_3_), 1.37–1.34 (t, *J* = 7.12 Hz, 3H, CH_3_CH_2_). ^13^C-NMR (100 MHz, DMSO-d_6_) δ_C_: 172.3 (C=O), 164.7 (N-C=N), 154.8 (N=C-H), 157.9, 150.6, 147.8, 142.6, 139.5 (2 × CH), 135.7 (CH), 134.5 (CH), 130.8 (2 × CH), 128.2 (CH), 117.3 (CH), 61.8 (CH_2_), 22.9 (OCH_3_), 14.5 (CH_3_). DEPT 135 (100 MHz, DMSO-d_6_) δ_C_: +ve signals are: 154.8 (N=C-H), 139.5 (2 × CH), 135.7 (CH), 134.5 (CH), 130.8 (2 × CH), 128.2 (CH), 117.3 (CH), 22.9 (OCH_3_), 14.5 (CH_3_). -ve signal: 61.8 (CH_2_) ppm. UV-Vis.: λ_max_ (nm)/log ε_max_ (M^-1^ cm^-1^): 225 (4.49), 227 (4.63), 255 (4.65), 272 (5.06), 338 (4.92). IR (KBr, cm^-1^) ῡ: 3,050 (C-H aromatic), 1,682 (C=O), 1,602 (C=C aromatic), 1,572 (C=N), 1,383 (CH_3_ deformation), 1,300 (C-N of hydrazide), 1,116 (C-O, of OEt), 943 (=C-H bending), 748 (Ar-H).

##### 2.1.3.12 Synthesis of 3-((4-hydroxy-3-methoxybenzylidene)amino)-2-methylquinazolin-4(3*H*)-one 3L

Reaction of 3-amino-2-methylquinazolin-4(*3H*)-one 2 (0.50 g, 2.87 mmol) with vanillin (0.44 g, 2.87 mmol) gave cream-colored solid 3L (0.77 g, 86%, melting point = 100–102°C). ^1^H-NMR (400 MHz, DMSO-d_6_) δ_H_: 9.85 (s, 1H, N=C-H), 8.90 (s, 1H, Aryl-H), 8.64–8.63 (d, *J* = 5.24 Hz, 1H, Aryl-H), 8.48–8.46 (d, *J* = 8.32 Hz, 1H, Aryl-H), 7.98–7.86 (d, *J* = 7.84 Hz, 1H, Aryl-H), 7.57–7.53 (dd, *J*_1_ = 6.36 Hz, *J*_2_ = 8.32 Hz, 1H, Aryl-H), 7.13–7.11 (dd, *J*_1_ = 6.36 Hz, *J*_2_ = 7.84 Hz, 1H, Aryl-H), 6.75–6.74 63 (d, *J* = 5.24 Hz, 1H, Aryl-H), 6.51 (s, 1H, OH), 3.43 (s, 3H, CH_3_), 2.15 (s, 3H, CH_3_). ^13^C-NMR (100 MHz, DMSO-d_6_) δ_C_: 172.5 (C=O), 159.5 (N-C=N), 156.1 (C-O), 153.8 (C-O), 152.1 (N=C-H), 145.8 (CH), 144.5, 135.8 (CH), 133.4, 130.2 (CH), 128.9, 123.1 (CH), 119.3 (CH), 117.1 (CH), 111.4 (CH), 45.5 (OCH_3_), 24.1 (CH_3_) ppm. DEPT 135 (100 MHz, DMSO-d_6_) δ_C_: +ve signals: 152.1 (N=C-H), 145.8 (CH), 135.8 (CH), 130.2 (CH), 123.1 (CH), 119.3 (CH), 117.1 (CH), 111.4 (CH), 45.5 (OCH_3_), 24.1 (CH_3_) ppm. -ve signals: nil.

##### 2.1.3.13 Synthesis of 2-methyl-3-((thiophen-2-ylmethylene)amino)quinazolin-4(3*H*)-one 3 m

Reaction of 3-amino-2-methylquinazolin-4(*3H*)-one **2** (0.50 g, 2.87 mmol) with thiophene-2-carbaldehyde (0.27 mL, 2.87 mmol) gave brown-colored solid 3m (0.49 g, 66%, melting point = 170–172°C). ^1^H-NMR (400 MHz, DMSO-d_6_) δ_H_: 9.93 (s, 1H, N=C-H), 8.49–8.47 (d, *J* = 8.40 Hz, 1H, Aryl-H), 7.97–7.95 (d, *J* = 7.92 Hz, 1H, Aryl-H), 7.72–7.70 (d, *J* = 6.44 Hz, 1H, Thiophene-H), 7.56–7.52 (dd, *J*_1_ = 8.40, *J*_2_ = 15.20 Hz, 1H, Aryl-H), 7.31–7.29 (d, *J* = 9.16 Hz, 1H, Thiophene-H), 7.12–7.08 (t, *J*_1_ = 7.92 Hz, *J*_2_ = 15.20 Hz, 1H, Aryl-H), 6.76–6.74 (m, 1H, Thiophene-H), 2.14 (s, 3H, CH_3_). ^13^C-NMR (100 MHz, DMSO-d_6_) δ_C_: 171.9 (C=O), 166.4 (N-C=N), 157.3 (N=C-H), 155.6, 137.1 (CH), 136.5, 131.3 (CH), 129.5 (CH), 128.9 (CH), 128.7 (CH), 127.7, 121.5 (CH), 117.5 (CH), 23.7 (CH_3_) ppm. DEPT 135 (100 MHz, DMSO-d_6_) δ_C_: +ve signals: 157.3 (N=C-H), 137.1 (CH), 131.3 (CH), 129.5 (CH), 128.9 (CH), 128.7 (CH), 121.5 (CH), 117.5 (CH), 23.7 (CH_3_) ppm. -ve signals: nil. IR (KBr, cm^-1^) ῡ: 3,050 (C-H aromatic), 2,929 (C-H aliphatic), 2,855 (C-H aliphatic), 1,682 (C=O), 1,615 (C=C), 1,577 (C=N), 1,393 (CH_3_ deformation), 923 (=C-H), 749 (Ar-H).

### 2.2 *In silico* studies

#### 2.2.1 Protein structures and preparation

The three-dimensional crystal structures of all the protein targets used for both the antibacterial and the antifungal *in silico* studies were obtained from the RCSB Protein Data Bank (Burley et al., 2019), with the exception of *Rhizopus nigricans* phosphotransferase, which was retrieved as a homology-modeled structure from AlphaFold (AF) (Waterhouse et al., 2018) (Table 1). The crystal structures of the proteins retrieved from the PDB were experimentally validated via x-ray crystallography (Qi et al., 2021). The resolutions of the targets selected for the study ranged from 1.37 Å to 3.80 Å, indicating that they were mostly high-resolution structures (Sharma et al., 2016). The protein structures were prepared using the Chimera tool by removing water molecules and non-amino acid residues, as well as adding hydrogen atoms. Minimization was performed using the AMBER ff14SB forcefield (Pettersen et al., 2004).

**TABLE 1:** Details of the protein targets used in the study, as obtained from Protein Data Bank.

S/N	Biological activities	Organisms	Protein target	PDB ID	Resolution Å)
**1**	Antibacterial activity	*Streptococcus mutans*	Sortase A	4TQX	1.37
		*Staphylococcus aureus*	DNA gyrase	2XCS	2.10
		*Escherichia coli*	Glucosamine-6-phosphate synthase	2VF5	2.90
		*Salmonella typhimurium*	Outer membrane protein F	4KR4	3.80
**2**	Antifungal activity	*Candida albicans*	N-myristoyltransferase	1IYL	3.20
		*Aspergillus niger*	3-phytase A	3K4Q	2.20
		*Aspergillus flavus*	Glucose oxidase, putative	4YNT	1.78
		*Rhizopus nigricans*	Phosphotransferase	(AF ID: AFA0A367KUY9)	-

#### 2.2.2 Ligand preparation

The two-dimensional structures of the synthesized substituted arylidene-based quinazolin-4(3*H*)-one motifs were drawn using the ChemDraw software package (Cousins, 2005). The compound structures were retrieved in SMILES format and used as inputs to the Online SMILES Translator and Structure File Generator of the NCI/CADD Group (Oellien and Nicklaus, 2004) for conversion to 3D structures. The 3D structures of the compounds were minimized using the universal force field (uff) function in PyRx software to prepare them for the docking analyses (Dallakyan and Olson, 2015).

#### 2.2.3 Molecular docking studies and post-docking analyses

Molecular docking studies were carried out using the AutoDock vina tool (Trott and Olson, 2009) to examine the binding conformations and affinities of the prepared ligands (3a–m) to the active site residues of the protein targets. The vina search spaces for the docking analyses were fitted around the active site residues of the protein, with an exhaustiveness value of 8. Post-docking analyses were carried out using the Discovery Studio visualizer to determine the protein–ligand binding interactions.

#### 2.2.4 Molecular dynamics (MD) simulation

The best hits identified on the basis of the *in silico* docking results were subjected to MD simulation to determine the stability of the compounds in docking models. MD simulation was performed using NAMD (Nanoscale Molecular Dynamics) (Phillips et al., 2005) and VMD (Visual Molecular Dynamics) (Humphrey et al., 1996). The RMSD (root mean square deviation) of the best hits and the c-alpha of the protein backbone in the protein–ligand complexes were examined. Energy minimization was carried out using 1,000 steps to fix the backbone atoms, while the production simulation run was carried out for 1 ns (equivalent to 10,020 frames). The simulation was conducted at constant pressure (1 atm) and temperature (310 K) with periodic boundary conditions. The protein structure file (PSF) of each target was generated separately from the ligands using VMD, while those of ligands were generated using the Charmm36 forcefield of the Charmm-GUI webserver. These were created to define the bond types, bond angles, atom types, and number of molecules in the simulation system. The topologies (PDB and PSF) of the proteins and ligands were merged, and the complexes were solvated using VMD to generate cubic water boxes. The other necessary parameters for the simulation (time and periodic boundary conditions) were defined in a script and run using NAMD. Graphs were obtained using the plot () function of the R programming software package.

### 2.3 Experimental antimicrobial screening

*In vitro* antibacterial and antifungal screening was performed to validate the results obtained in the *in silico* study. Testing of the general sensitivity and minimum inhibitory concentration (MIC) of the synthesized arylidene-based quinazolin-4(3*H*)-one motifs was carried out using agar diffusion and serial dilution methods, respectively. MIC experiments were carried out in triplicate. *In vitro* antimicrobial screening was conducted using a previously established method (see Supplementary Material) (Ajani et al., 2010). The microorganisms used for screening included two Gram-positive bacteria (*Streptococcus mutans* and *Staphylococcus aureus*), two Gram-negative bacteria (*E. coli* and *Salmonella typhimurium*), and four fungi (*C. albicans*, *A. niger*, *A. flavus*, and *R. nigricans*). The selection of these specific microorganisms was grounded in their significance to human health and the need to evaluate the potential antimicrobial efficacy of the synthesized compounds against these organisms. Among the bacterial species, *S. mutans* holds importance due to its role in dental health and tooth decay (Krzyściak et al., 2017); *S. aureus* is a versatile pathogen causing diverse infections, from skin issues to severe conditions like sepsis (Morsy et al., 2020); *Escherichia coli* typically resides in the gut and can trigger food poisoning (Kant et al., 2016); and *S. typhimurium* is a common culprit in foodborne illnesses (Campos et al., 2019). *Candida albicans* is a yeast-like fungus causing infections in vulnerable individuals, while *Aspergillus niger* is linked to respiratory infections in immunocompromised individuals (Morsy et al., 2020). *Aspergillus flavus* is known for producing toxic compounds and for food contamination, while *R. nigricans* is a mold that occasionally affects immunocompromised individuals (Liu et al., 2018).

### 2.4 Predicted ADMET studies

The drug-likeness, medicinal chemistry, and pharmacokinetic properties of the substituted benzylidene-based quinazolin-4(3*H*)-one (3a–l) and the thiophenylidene-based quinazolin-4(3*H*)-one (3m) motifs were predicted using ADMETLAB (Xiong et al., 2021) and SWISSADME (Daina et al., 2017). These predictions aimed to suggest the potential absorption, distribution, metabolism, excretion and toxicity (ADMET) properties of the compounds. The properties predicted/determined included number of rotatable bonds; hydrogen bond acceptor; hydrogen bond donor; molar refractivity; topological polar surface area (TPSA); lipophilicity; a measure of drug-likeness (QED); synthetic accessibility; conformance to the Lipinski rule, Pfizer rule, and GSK rule; gastrointestinal absorption; blood brain barrier (BBB) permeant status; and P-glycoprotein substrates.

## 3 Results and discussion

### 3.1 Chemistry

**SCHEME 1:**
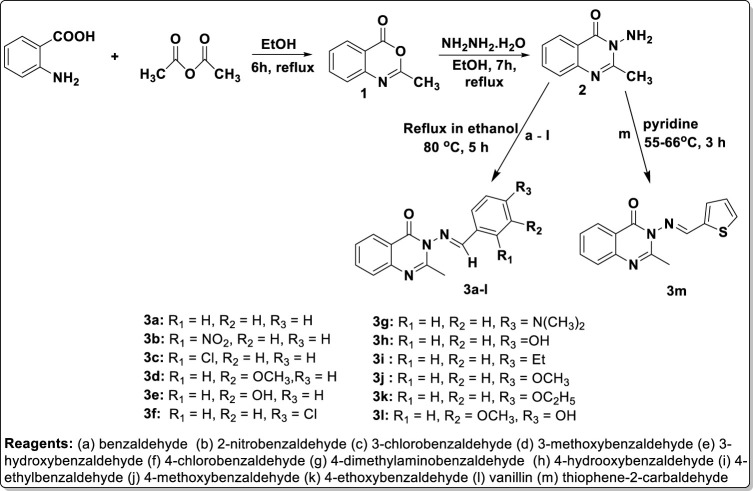
Synthesis of the arylidene-based quinazolin-4(3*H*)-one analogs, 3a–m.

Nitrogen-containing heterocyclic motifs are valuable templates in drug development. The present study represents the advancement of our efforts in both the synthesis of and exploration of the biological potential of *N*-heterocyclic compounds (Ajani et al., 2017b; 2017c; 2018). Initially, the thermal annelation of acetic anhydride onto anthranilic acid was effectively achieved at 85°C in ethanol after 6 h of conventional reflux, resulting in the formation of precursor 1 with a yield of 95%, using our previously reported procedure (Ajani et al., 2017b). Hydrazinolysis of the synthesized precursor 1 led to the structural modification via nitrogen insertion at its lactone portion, yielding amino-containing intermediate 2 with a yield of 98%. This was followed by condensation of the free amino group in intermediate 2 with benzaldehyde a) and eleven other substituted benzaldehydes b) to (l), achieved through refluxing at 80°C for up to 5 h, resulting in the formation of compounds 3a–l. The reaction completion was confirmed by TLC monitoring (eluent: CH_2_Cl_2_), and the final targeted products were substituted benzylidene-based quinazolin-4(3*H*)-one analogs 3a–l, obtained at yields ranging from 46% to 99% (Scheme 1). The work-up was achieved by evaporating the solvent using a rotary evaporator, and the solid mass of crude products obtained was purified through recrystallization in ethanol, yielding pure products 3a–l. Additionally, thiophene was formylated via a Vilsmeier–Haack reaction using dimethylformamide (DMF) in phosphoryl oxychloride at 0°C, resulting in the production of thiophene-2-carbaldehyde m), according to a known procedure (Mphahlele and Mmonwa, 2019). Thiophene-2-carbaldehyde m) was then nucleophilically attached by intermediate 2 in a condensative manner in pyridine, yielding thiophenylidene-based quinazolin-4(3*H*)-one analog 3m (Scheme 1).

Physicochemical characterization of the targeted products revealed that the molecular mass of precursor 1 (161.16 g/mol) was lower than that of intermediate 2 (175.19 g/mol), which in turn was lower than those of the final products 3a–m (257.31–309.33 g/mol). Considering the molecular weights of the synthesized compounds and their chemical structures, it can be inferred that these compounds comply with one of the criteria of the Lipinski rule (the rule of five), as their molecular weights do not exceed 500 g/mol (Chagas et al., 2018). Compounds 1 and 2 were obtained with yields of 95% and 98%, respectively, while the final products were obtained with yields ranging from 46% for compound 3j to 99% for 3e. All final motif products were achieved with encouragingly high yields, with the exceptions of compounds 3j (46%) and 3k (46%), which were obtained with yields below 50%. This lower yield in the cases of 3j and 3k may be attributed to a very low rate of crystallinity observed during the formation of these compounds. The melting point of precursor 1 was 171–173°C, and there was a sharp increase in melting point to 224–226°C for intermediate amino-containing compound 2. This confirms the effective conversion of the less polar lactone functionality of precursor 1 to the more polar 3-amino-containing cyclic amide template of compound 2. The melting points of the final quinazolin-3(4*H*)-one motifs 3a–m ranged from 100 to 102°C for 3L, the lowest melting point, to 285°C for 3a, the highest. Since 3a, with the highest melting point, contained a non-substituted benzylidene, it can be concluded that the presence of a substituent on the aromatic ring of the benzylidene portion reduces the melting point of the parent quinazolin-3(4*H*)-one motifs to which it belongs. Virtual observation showed that the colors of products 3a–m could be categorized into four main groups: brown for 3a, 3f, 3i, 3j, and 3m; yellow for 3b; cream for 3c–e, 3h, 3k, and 3l; and orange for 3g.

**FIGURE 1:**
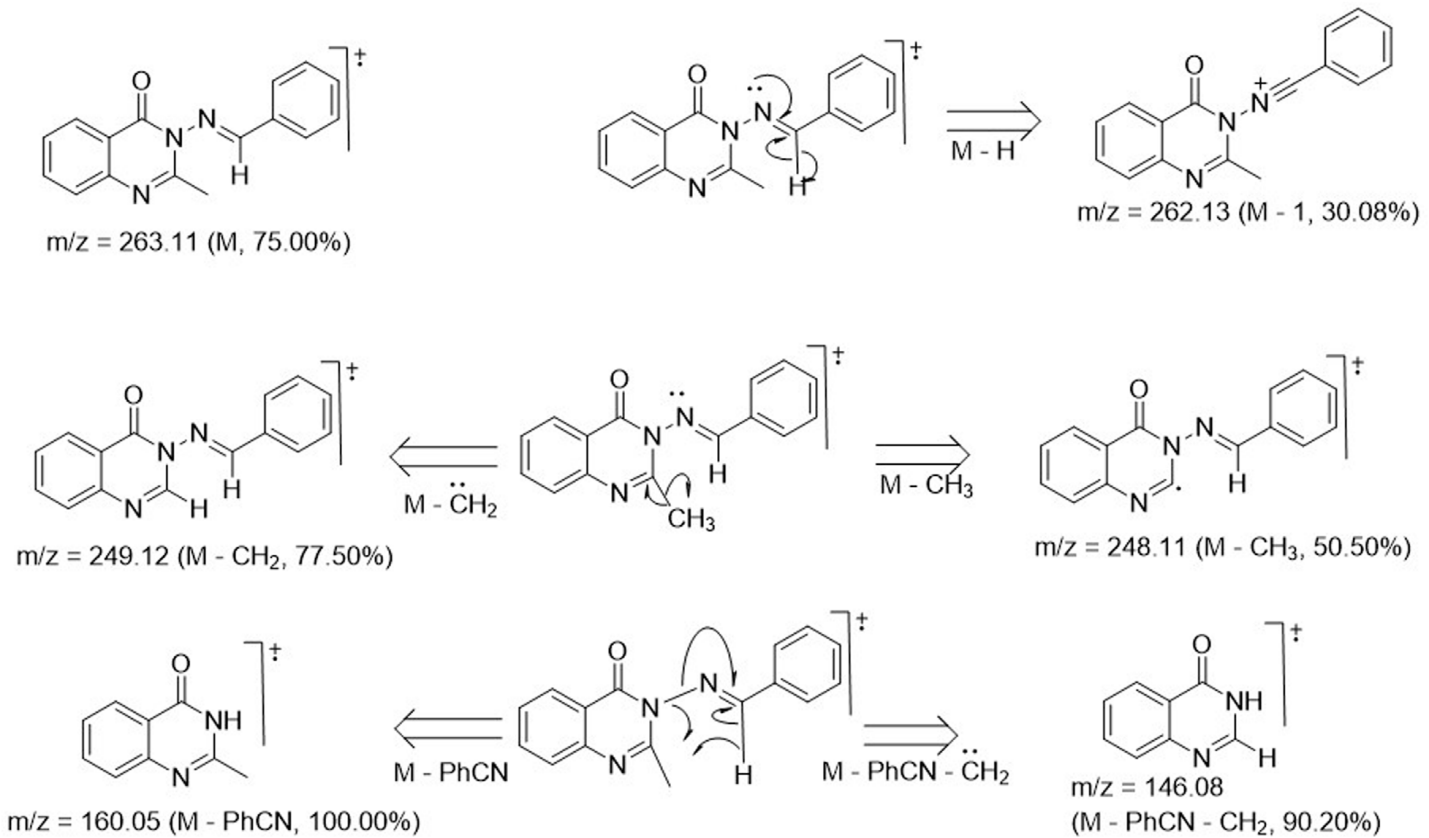
Images depicting fragmentation pattern for formation of daughter ions.

Structural elucidation and validation of the synthetic analogs 3a–m was successfully achieved through spectral data generated from ^1^H and ^13^C NMR, DEPT-135 NMR, UV-visible spectroscopy, FT-IR, and mass spectrometry. Compound 3a was used as a representative of the series, and its spectroscopic features are discussed below. The ^1^H-NMR spectrum of compound 3a exhibited a 3H singlet at 2.12 ppm, which stood for the presence of CH_3_ at position 2 of the quinazolin-4(3*H*)-one ring. All other signals were seen further downfield, below 6.00 ppm. A 3H multiplet was observed at δ_H_ 6.76–6.67 ppm, indicating the presence of 3H from benzylidene moiety, while the remaining 2H located at the ortho-position of the benzylidene were chemically equivalent, manifesting as a doublet with δ_H_ 7.75–7.72 ppm and a coupling constant of 8.40 Hz. All 4H of the benzo-fused quinazoline ring resonated as a 1H doublet, each at δ_H_ 7.99–7.97 ppm (*J* = 6.48 Hz); a 1H doublet at δ_H_ 8.52–8.50 ppm (*J* = 6.82 Hz); and a 1H multiplet, each at δ_H_ 7.56–7.51 ppm and 7.12–7.08 ppm. The most deshielded proton was that of azomethine (N=C-H), which resonated as the most downfield signal, observed as a 1H singlet at δ_H_ 9.72 ppm. The ^13^C-NMR run at 100 MHz exhibited 16 carbon atoms, varying from 172.7 ppm for C=O in the case of quinazolin-4(3*H*)-one to 22.5 ppm for methyl on the 2-position of the quinazolinone ring. These values were consistent with the proposed structure, and the signal values were as expected. The DEPT 135 NMR spectrum of 3a revealed no negative signals, indicating the absence of methylene (CH_2_) groups in the structure of 3a. Instead, nine positive signals were observed, consisting of nine methine (CH) carbon ranging from 154.4 to 110.7 ppm, along with one methyl (CH_3_) carbon at 22.5 ppm. This observation was consistent with the total number of CH and CH_3_ carbon atoms of 3a. The FT-IR spectrum of 3a displayed stretching frequencies at 3,020 cm^-1^, 2,824 cm^-1^, 1,676 cm^-1^, 1,602 cm^-1^, and 1,581 cm^-1^, which corresponded to diagnostic bands indicating the presence of CH in the aromatic ring, CH in the aliphatic region, carbonyl in the quinazolinone, C=C in the aromatic, and C=N in the quinazoline ring, respectively. The CH character in the aliphatic region was further confirmed to arise from the methyl group, as evidenced by the bending vibrational mode at 1,376 cm^-1^, representing CH_3_ deformation. Additionally, the aromatic character was doubly confirmed in the fingerprint region by the presence of bending vibrational bands at 980 cm^-1^, 869 cm^-1^, and 747 cm^-1^, corresponding to = C-H, C=C (out-of-plane bending), and Ar-H (bending and ring puckering), respectively. The mass spectrum of compound 3a is presented in Figure 1. The molecular ion peak accounted for the m/z value of 263.11, with relative abundance of 75%. This value was consistent with the molecular weight of analog 3a, confirming an error of 0 ppm. This alignment indicated that the proposed structure for 3a was accurate. The base peak was observed at m/z of 160.05, with an intensity of 100%. This peak represented the stable molecule formed after the loss of the benzonitrile functionality from compound 3a. The fragmentation pattern is illustrated in Figure 1. The daughter ions at m/z 249.12 (with an intensity of 77.50%) and m/z 248.11 (with an intensity of 50.50%) indicated the loss of methylene (CH_2_) and methyl (CH_3_) units, respectively, from 3a due to high electron bombardment in the spectrometer chamber. Additionally, the fragment at m/z 146.08 (with an intensity of 90%) resulted from the loss of benzonitrile and a methylene unit.

### 3.2 *In silico* antibacterial studies

#### 3.2.1 Molecular docking studies

The binding affinities and conformations of the substituted arylidene-based quinazolin-4(3*H*)-one motifs 3a–m in the active sites of the antibacterial protein targets (*S. mutans* sortase A, *S. aureus* DNA gyrase, *E. coli* glucosamine-6-phosphate synthase, and *S. typhimurium* outer membrane protein F) were determined through molecular docking studies. Docking analyses of protein–ligand interactions for the predicted antibacterial activities indicated favorable binding affinities of the compounds (3a–m) with the targets (Table 2). Compound 3l exhibited the lowest binding energy of -7.0 kcal/mol for *S. mutans* sortase A (PDB ID: 4TQX), followed by compound 3g, with a binding affinity of −6.9 kcal/mol. In the case of *S. aureus* gyrase (PDB ID: 2XCS), compounds 3c and 3e had the best binding affinities, each with binding energies of −9.6 kcal/mol; however, these were not better than the cofactor RXV (−10.0 kcal/mol). Compound 3l possessed the lowest binding energy for *E. coli* glucosamine-6-phosphate synthase (PDB ID: 2VF5) with a value of −8.0 kcal/mol, better than the cofactor GLP (−7.3 kcal/mol). Furthermore, compound 3i possessed the lowest binding energy, with a value of −9.0 kcal/mol, for *S. typhimurium* outer membrane protein F (PDB ID: 4KR4), followed by compound 3h, with a binding affinity of −8.5 kcal/mol; both compounds had better binding affinities than the cofactor AIC (−6.9 kcal/mol). The better binding affinities to the targets observed for some of the compounds in comparison to the cofactors suggest that the compounds are better binders to the active sites of the targets (Rakesh et al., 2019). Notably, all best hits for each organism represented better binding affinities than the standard gentamicin used.

**TABLE 2:** Binding affinities of the substituted quinazolin-4(3*H*)-one motifs in the active sites of the 3D structures of the antibacterial protein targets.

Sample code	*S. mutans* (4TQX) {kcal/mol}	*S. aureus* (2XCS) {kcal/mol}	*E. coli* (2VF5) {kcal/mol}	*S. typhimurium* (4KR4) {kcal/mol}
**3a**	−6.5	−9.5	−7.5	−8.2
**3b**	−6.9	−9.4	−7.5	−7.8
**3c**	−6.5	−9.6	−7.6	−7.8
**3d**	−6.6	−9.5	−7.5	−8.2
**3e**	−6.8	−9.6	−7.5	−8.3
**3f**	−6.7	−9.0	−7.6	−8.5
**3g**	−6.9	−8.9	−7.6	−7.9
**3h**	−6.9	−9.0	−7.5	−8.5
**3i**	−6.9	−9.1	−7.6	−9.0
**3j**	−6.7	−9.3	−7.4	−8.3
**3k**	−6.6	−9.0	−7.3	−7.9
**3l**	−7.0	−9.4	−8.0	−8.5
**3m**	−5.4	−8.3	−6.3	−6.3
**Gentamicin**	−6.6	−9.4	−7.7	−7.9
**Cofactor**	NONE	RXV (−10.0)	GLP (−7.3)	AIC (−6.9)

RXV: 6-methoxy-4-(2-{4-[([1,3]oxathiolo[5,4-c]pyridin-6-ylmethyl)amino]piperidin-1-yl}ethyl)quinoline-3-carbonitrile; GLP: 2-amino-2-deoxy-6-O-phosphono-alpha-D-glucopyranose; AIC (2S,5R,6R)-6-{[(2R)-2-amino-2-phenylethanoyl]amino}-3,3-dimethyl-7-oxo-4-thia-1-azabicyclo[3.2.0]heptane-2-carboxylic acid.

**FIGURE 2:**
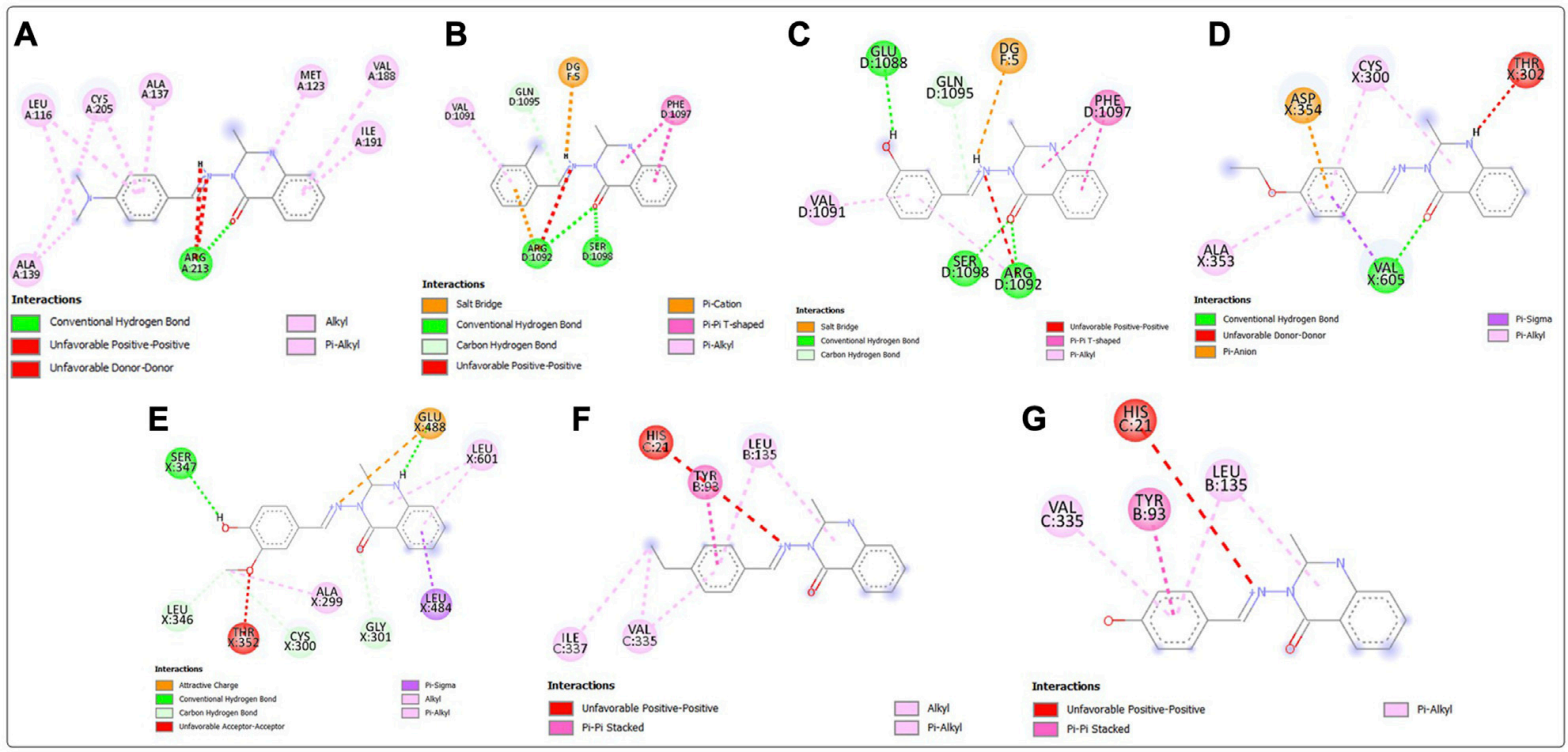
Post-docking analyses showing the 2D interactions between the best hits and active sites of the protein target for antibacterial activities **(A)** 3g and 4TQX **(B)** 3c and 2XCS **(C)** 3e and 2XCS **(D)** 3k and 2VF5 **(E)** 3L and 2VF5 **(F)** 3i and 4KR4 **(G)** 3h and 4KR4.

Post-screening analyses revealed the possible atoms of the compounds and amino acid residues of the proteins responsible for the protein–ligand binding (Daddam et al., 2020). The interactions formed between the atoms of the best hits and the active site residues were examined, as shown in Figure 2. Predominant interactions in the protein–ligand complexes encompassed conventional hydrogen bonds, carbon hydrogen bonds, alkyl, pi-alkyl, pi-cation, pi-pi T-shaped, salt bridge, pi-sigma, pi-anion, and pi-pi stacking interactions. Unfavorable bond interactions were also observed, suggesting repulsive forces between molecules (Tallei et al., 2021). All these interactions play major roles in the strength of protein–ligand complexes and in the docking scores obtained for each of the best hits (Thillainayagam et al., 2017; Oduselu et al., 2023). Compound 3g formed a conventional hydrogen bond with ARG A213 of *S. mutans* sortase A (PDB ID: 4TQX) via the carbonyl oxygen on the quinazolinone core structure. Compounds 3c and 3e, which possessed the same binding affinity against *S. aureus* gyrase (PDB ID: 2XCS), also had similar hydrogen bond interactions with the active site of 2XCS. In this case, carbonyl oxygen on the quinazolinone core structure of compounds 3c and 3e formed conventional hydrogen bonds with ARG D1092 and SER D1098. However, compound 3e formed additional hydrogen bonds with GLU D1088 and GLN D1095. Compound 3k formed a hydrogen bond with VAL X605 of *E. coli* glucosamine-6-phosphate synthase (PDB ID: 2VF5) via the carbonyl oxygen on the quinazolinone core structure, while compound 3l formed a hydrogen bond with GLY X301 via the same oxygen atom. This suggests that the presence of the carbonyl oxygen on the quinazolinone template of the substituted benzylidene-based quinazolin-4(3*H*)-one motifs is very important in the binding interaction, since it is a good hydrogen bond acceptor (Sodero et al., 2017). No hydrogen bond was observed in the interactions of compounds 3i and 3h with the active site of *S. typhimurium* outer membrane protein F (PDB ID: 4KR4).

#### 3.2.2 Molecular dynamics simulation studies

**FIGURE 3:**
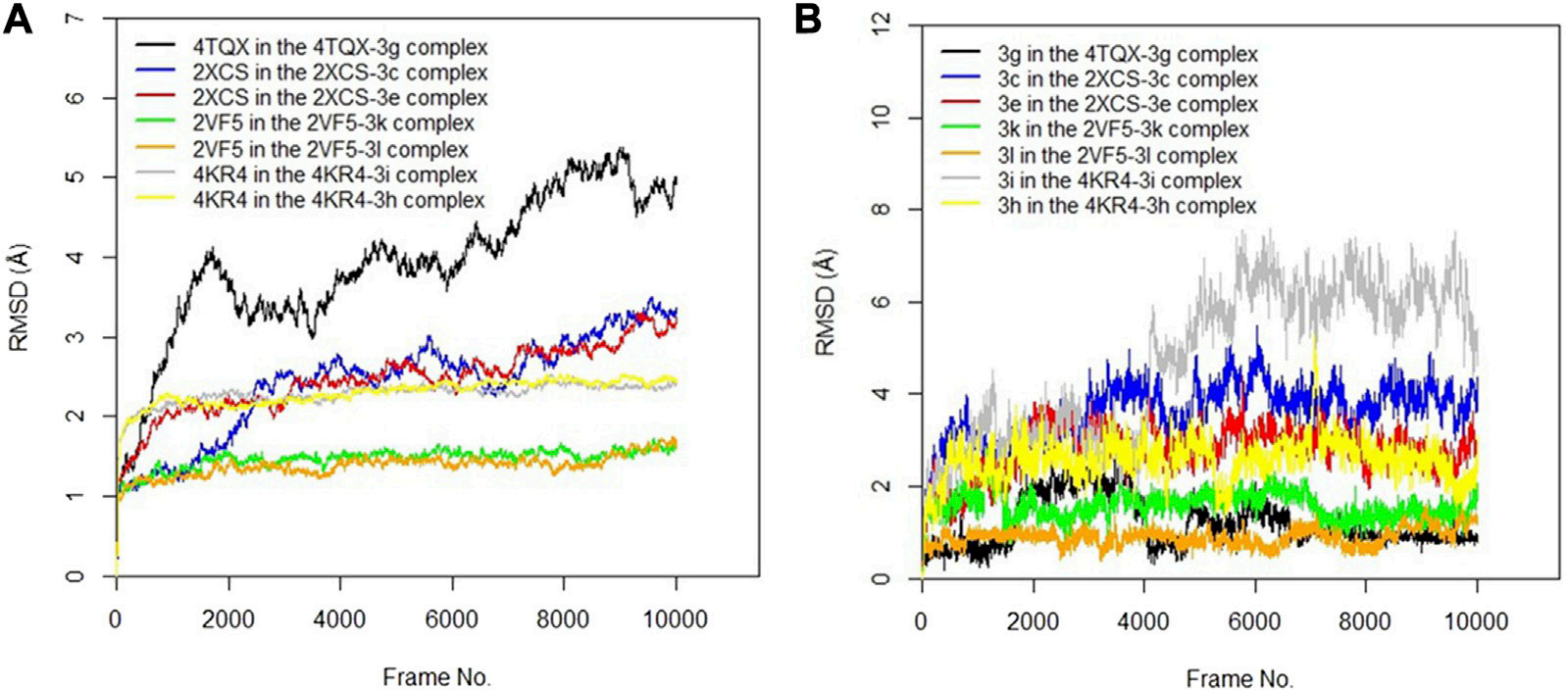
**(A)** RMSD time series for the c-alpha of the protein backbone for *S. mutans* sortase A (4TQX), *S. aureus* DNA gyrase (2XCS), *Escherichia coli* glucosamine-6-phosphate synthase (2VF5), and *S. typhimurium* outer membrane protein F (4KR4) in the complexes formed with the best hits from molecular docking studies **(B)** RMSD time series for the best hits from molecular docking studies (3g, 3c, 3e, 3k, 3l, 3i, and 3h) when complexed with the antibacterial protein targets.

The RMSD values of the protein–ligand complexes were analyzed via molecular dynamics simulation studies; these revealed the structural stability of the protein target in the complex (Surti et al., 2020). Figures 3A,B show RMSD plots of the c-alpha of the protein backbones and of the compounds in the protein–ligand complex. Significant changes, with fluctuations in RMSD by > 3, suggest instability of the ligand or the protein in the complex (Oduselu et al., 2023). The MD simulation revealed that all the protein targets for the predicted antibacterial activities had fluctuations of RMSD by < 3, except in the case of 4TQX when complexed with compound 3 g (Figure 3A). This suggests that the proteins were structurally stable throughout the simulation. Furthermore, the RMSD values of the compounds indicated their relatively stable conformations during the simulation, except in the case of 3i in the 4KR4-3i complex (Figure 3B). Among all protein–ligand complexes examined for predicted antibacterial activities, the 2VF5-3 k complex appeared to be the most stable. The c-alpha backbone for the protein exhibited an average RMSD value of 1.386 Å and a standard deviation of 0.130 Å, while compound 3k displayed an average RMSD value of 0.895 Å and a standard deviation of 0.191 Å (Supplementary Table S1). Overall, the results revealed that the formation of protein–ligand complexes did not compromise the structural stability of the proteins and ligands.

### 3.3 *In silico* antifungal studies

#### 3.3.1 Molecular docking studies

Docking analyses of protein–ligand interactions for the predicted antifungal activities revealed that the compounds (3a–m) exhibited good binding affinities with the targets (Table 3). Compound 3c had the lowest binding energy, of −9.7 kcal/mol, for *C. albicans* N-myristoyltransferase (PDB ID: 1IYL), surpassing the cofactor R64 with binding energy of −9.6 kcal/mol. Compounds 3c and 3h possessed the best binding energy, of −7.2 kcal/mol, for *A. niger* 3-phytase A (PDB ID: 3K4Q), outperforming the cofactor IHS with binding energy of −6.5 kcal/mol. Compound 3b was the best hit for *A. flavus* glucose oxidase, putative (PDB ID: 4YNT), with a binding affinity of −9.9 kcal/mol; however, it was not a better binder than the cofactor FDA, with a binding affinity of −10.9 kcal/mol. Additionally, compound 3b possessed the best binding affinity against homology modeled *R. nigricans* phosphotransferase (AF ID: AFA0A367KUY9). Ketoconazole standard was observed to be a better binder than the best hits against *C. albicans* N-myristoyltransferase (PDB ID: 1IYL) for *A. niger* 3-phytase A (PDB ID: 3K4Q) and *R. nigricans* phosphotransferase (AF ID: AFA0A367KUY9). However, ketoconazole displayed lower binding affinity when compared to the best hit for *A. flavus* glucose oxidase, putative (PDB ID: 4YNT).

**TABLE 3:** Binding affinities of the substituted quinazolin-4(3*H*)-one motifs in the active sites of antifungal protein targets.

Sample code	*C. albicans* (1IYL) {kcal/mol}	*A. niger* (3K4Q) {kcal/mol}	*A. flavus* (4YNT) {kcal/mol}	*R. nigricans* (AFA0A367KUY9) {kcal/mol}
**3a**	−9.4	−7.0	−9.2	−7.6
**3b**	−9.6	−6.9	−9.9	−7.9
**3c**	−9.7	−7.2	−9.6	−7.8
**3d**	−9.3	−6.5	−8.7	−7.4
**3e**	−9.3	−6.9	−9.1	−7.6
**3f**	−9.2	−6.7	−9.3	−7.4
**3g**	−8.7	−6.6	−7.4	−7.5
**3h**	−9.5	−7.2	−9.4	−7.6
**3i**	−9.5	−6.6	−9.5	−7.6
**3j**	−9.1	−6.9	−9.1	−7.3
**3k**	−9.1	−6.3	−9.0	−7.3
**3l**	−9.1	−6.8	−9.4	−7.3
**3m**	−7.9	−5.6	−7.8	−6.4
**Ketoconazole**	−9.9	−7.3	−8.7	−8.9
**Control ligand**	R64 (−9.6)	IHS (−6.5)	FDA (−10.9)	NONE

R64 (1-methyl-1h-imidazole-2-yl)-(3-methyl-4-{3-[(pyridin-3-ylmethyl)-amino]-propoxy}-benzofuran-2-yl)-methanone; HIS: D-myo-inositol-hexasulphate; FDA: dihydroflavine-adenine dinucleotide.

**FIGURE 4:**
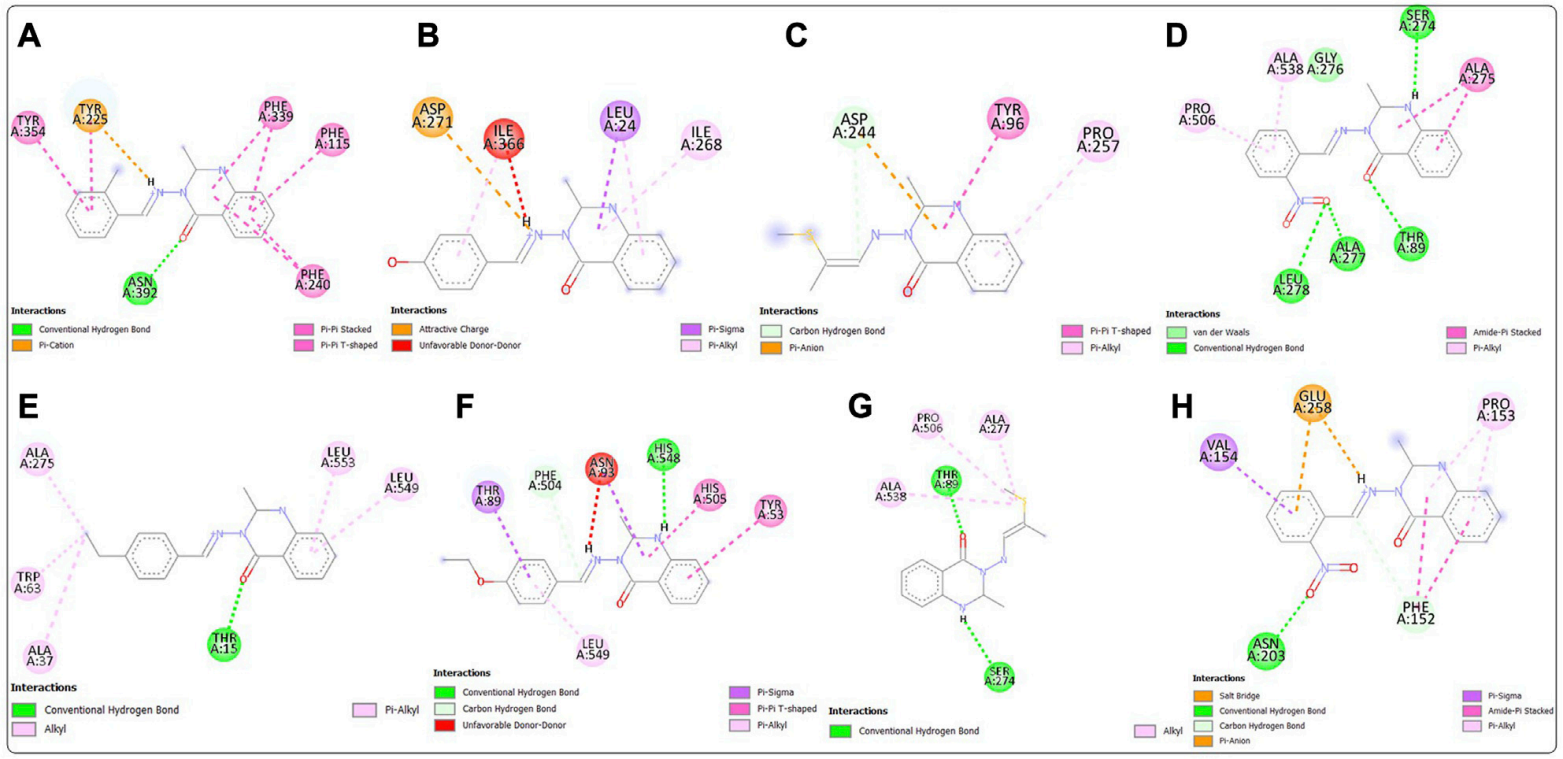
Post-docking analyses showing the 2D interactions between the best hits and active sites of the protein target for predicted antifungal activities **(A)** 3c and 1IYL **(B)** 3h and 3K4Q **(C)** 3m and 3K4Q **(D)** 3b and 4YNT **(E)** 3i and 4YNT **(F)** 3k and 4YNT **(G)** 3m and 4YNT **(H)** 3b and AFA0A367KUY9.

Post-docking analyses for the predicted antifungal activities revealed the interactions between the molecules and the amino acid residues present in the active sites of the protein targets (Figure 4). The presence of the hydrogen bond donors and hydrogen bond acceptors found on the compound structures enabled the formation of the hydrogen bonds observed in the protein–ligand complexes (Oduselu et al., 2021b). The carbonyl oxygen of the benzylidene-based quinazolin-4(3*H*)-one motif 3c formed a conventional hydrogen H) bond with ASN A392 residue of *C. albicans* N-myristoyltransferase (PDB ID: 1IYL), among many other molecular interactions. The highest number of hydrogen bond interactions was observed in the complex formed between compound 3b and *A. flavus* glucose oxidase, putative (PDB ID: 4YNT). In this case, the carbonyl oxygen of the quinazolinone template formed an H bond with THR A89, and the secondary amine hydrogen of the quinazolinone template formed an H bond with SER A 274. Additionally, one of oxygen atoms of the nitro group on position 2 of the substituted benzylidene group on compound 3b formed hydrogen bonds with LEU A278 and ALA A277. Remarkably, compound 3b, with a total of four H bonds, exhibited the strongest binding affinity among the docked 4YNT complexes. Compounds 3i and 3k formed one hydrogen bond each with THR A15 and HIS A548 amino acid residues of *A. flavus* glucose oxidase, putative (PDB ID: 4YNT), respectively. Moreover, only one hydrogen bond was observed between compound 3b and the ASN A203 residue of homology modeled *R. nigricans* phosphotransferase (AF ID: AFA0A367KUY9).

#### 3.3.2 Molecular dynamics simulation

**FIGURE 5:**
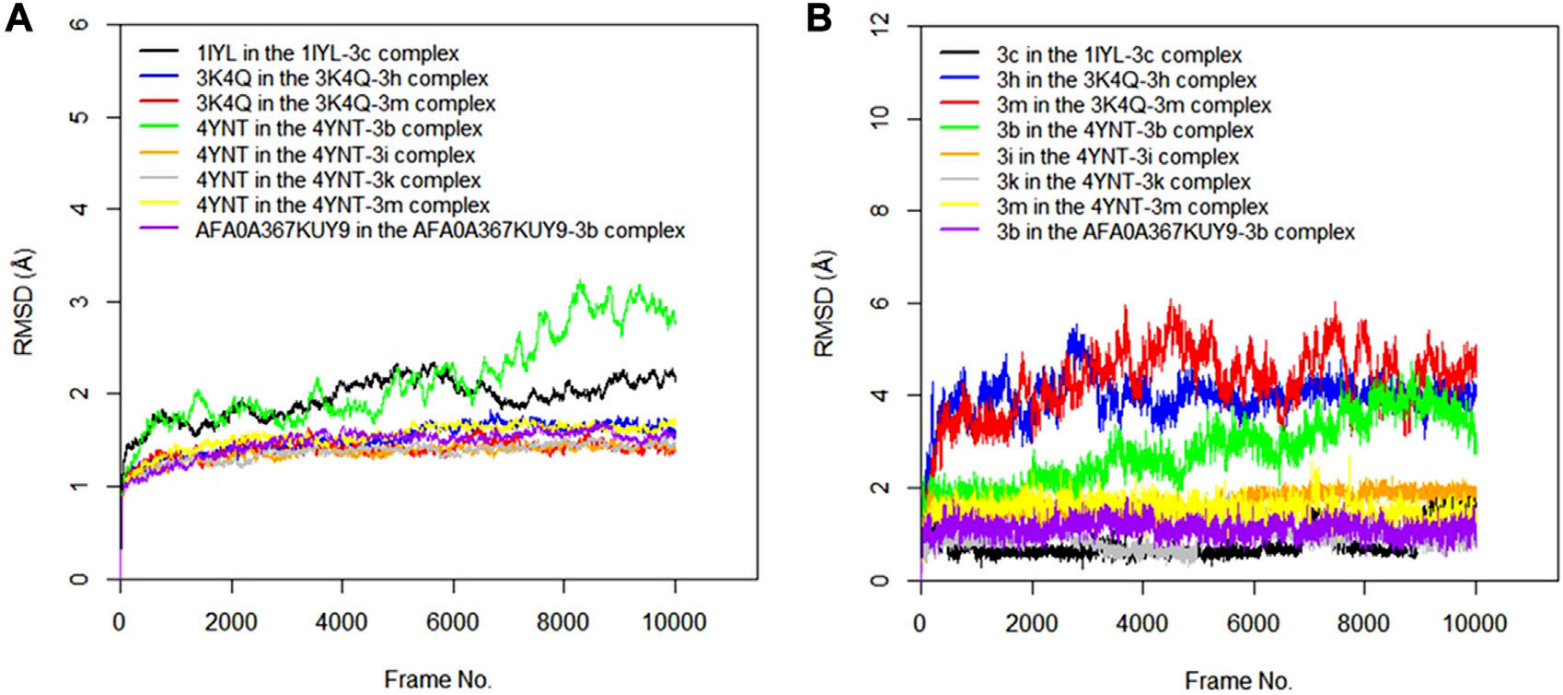
**(A)** RMSD time series for the c-alpha of the protein backbone for *Candida albicans* N-myristoyltransferase (PDB ID: 1IYL), *Aspergillus niger* 3-phytase A (PDB ID: 3K4Q), *Aspergillus flavus glucose oxidase, putative* (PDB ID: 4YNT), and *Rhizopus nigricans* phosphotransferase (AF ID: AFA0A367KUY9) in the complexes formed with the best hits from molecular docking studies **(B)** RMSD time series for the best hits from molecular docking studies (3c, 3h, 3m, 3b, 3i, and 3k) when complexed with the antifungal protein targets.

The RMSD values of the protein–ligand complexes for the predicted antifungal activities were analyzed via molecular dynamics simulation studies. Figures 5A,B show RMSD plots illustrating the stability levels of the c-alpha of the protein backbones and of the compounds in the target ligand complex. MD simulation outcomes demonstrated that all the protein targets for the predicted antifungal activities displayed RMSD fluctuations by < 3.00 Å after an initial rise from 0.00 Å, indicating the stability of the protein targetin the complex (Figure 5A). Similar trends were observed for the compounds examined via MD simulation, with all ligands maintaining relatively stable conformations throughout the simulation, with RMSD <3.00 Å (Figure 5B). Overall, all the ligands and protein targets monitored during the MD simulation showed excellent levels of structural stability, as evidenced by the slight variation observed in their RMSD values after 10,020 frames.

### 3.4 Experimental validation of antimicrobial activities

The antimicrobial potential of the quinazolin-4(3*H*)-one motifs 3a–m was determined via *in vitro* screening against four strains of bacteria, comprising three type-cultured organisms (*S. aureus* (ATCC 25923), *E. coli* (ATCC 25922), and *S. typhimurium* (ATCC 14028)) and one locally isolated organism (*S. mutans* (LIO)), using the agar well diffusion method. The activities of the prepared motifs 3a–m were measured in comparison to that of gentamicin, a clinical antibacterial standard. The results of general sensitivity testing are shown in Table 4, indicated in the form of zones of inhibition (ZOIs) measured in millimeters (mm). The choice of *S. mutans* for testing in the present study was made due to its wide occurrence in dental caries, which is a chronic disease among the human population and is recognized as the most common childhood disease (Krzyściak et al., 2017). The synthesized compounds exhibited significant activity, with large ZOIs, against all four bacterial strains, except in three instances. These were the noticeable resistance of all four bacterial strains to motif 3b, the resistance of *S. mutans* to compound 3j, and the resistance of the remaining three bacterial strains (*S. aureus*, *E. coli*, and *S. typhimurium*) to the growth-inhibitory potential of 3g. The ZOIs of compounds 3a–m against *S. mutans* ranged from 15.00 ± 0.40 mm to 35.00 ± 0.98 mm, while the ZOI of the gentamicin standard was 24.00 ± 0.60 mm (Table 4). All active compounds, except 3g, exhibited larger ZOIs than the gentamicin standard. Hence, they could be considered to be enhancers of the bioactivity of gentamicin in treating tooth decay, given that *S. mutans* is the bacterial stain responsible for dental caries, commonly known as the tooth decay (Krzyściak et al., 2017). The antibacterial activities of compounds 3a–m showed the largest ZOIs against *S. aureus*, ranging from 40.00 ± 1.12 mm to 50.00 ± 1.34 mm, nearly double that of gentamicin (22.00 ± 0.60 mm) against *S. aureus*, which is a bacterium associated with high mortality rates among humans, leading to increased hospital admissions and substantial financial implications relating to treatment (Nwinyi et al., 2008).

**TABLE 4:** Results of *in vitro* antibacterial screening, in the form of zones of inhibition (ZOIs; mm), and minimum inhibitory concentration (MIC) testing on bacterial isolates.

S/N	Sample code	*S. mutans*		*S. aureus*		*E. coli*		*S. typhimurium*	
		ZOI (mm)	MIC (μg/mL)	ZOI (mm)	MIC (μg/mL)	ZOI (mm)	MIC (μg/mL)	ZOI (mm)	MIC (μg/mL)
1	**3a**	35.00 ± 0.90	62.50	50.00 ± 1.30	15.62	38.00 ± 1.10	31.25	30.00 ± 0.90	62.50
2	**3b**	R	N.D.	R	N.D.	R	N.D.	R	N.D.
3	**3c**	25.00 ± 0.62	62.50	50.00 ± 1.33	3.90	25.00 ± 0.63	125.00	23.00 ± 0.60	62.50
4	**3d**	30.00 ± 0.90	31.25	40.00 ± 1.10	31.25	40.00 ± 1.10	31.25	35.00 ± 0.96	62.50
5	**3e**	33.00 ± 0.90	62.50	40.00 ± 1.10	3.90	30.00 ± 091	62.50	30.00 ± 0.90	62.50
6	**3f**	30.00 ± 0.90	15.62	40.00 ± 1.12	15.62	42.00 ± 1.15	62.50	30.00 ± 0.90	125.00
7	**3g**	15.00 ± 0.40	3.90	R	N.D.	R	N.D.	R	N.D.
8	**3h**	35.00 ± 0.96	7.81	50.00 ± 1.30	15.62	30.00 ± 0.90	31.25	35.00 ± 0.98	7.81
9	**3i**	30.00 ± 0.90	62.50	45.00 ± 1.12	31.25	40.00 ± 1.10	62.50	25.00 ± 0.62	31.25
10	**3j**	32.00 ± 0.90	15.62	45.00 ± 1.12	7.81	42.00 ± 1.12	7.81	28.00 ± 0.88	62.50
11	**3k**	35.00 ± 0.98	7.81	45.00 ± 1.13	3.90	45.00 ± 1.14	3.90	30.00 ± 0.91	15.62
12	**3l**	R	N.D.	40.00 ± 1.10	15.62	35.00 ± 0.96	31.25	30.00 ± 0.91	31.25
13	**3m**	25.00 ± 0.61	62.50	50.00 ± 1.34	1.95	25.00 ± 0.64	31.25	23.00 ± 0.61	31.25
14	**GTM**	24.00 ± 0.60	5.00	22.00 ± 0.60	1.25	25.00 ± 0.62	2.50	26.00 ± 0.62	1.25

Mean ± SD, standard deviation of triplicate measurements. S. mutans = Streptococcus mutans (LIO); S. aureus = Staphylococcus aureus (ATCC, 25923); E. coli = Escherichia coli (ATCC, 25922); S. typhimurium = Salmonella typhimurium (ATCC, 14028); ATCC, american type culture collection; LIO, locally isolated organism; GTM, gentamicin; N.D., not determined; ZOI, zone of inhibition; MIC, minimum inhibitory concentration.

In addition to the above results, susceptibility testing against *E.* coli, a Gram-negative bacterial isolate, revealed ZOIs ranging from 25.00 ± 0.64 mm to 42.00 ± 1.15 mm for compounds 3a–m, while the ZOI of gentamicin against *E. coli* was 25.00 ± 0.62 mm. These results indicated that quinazolin-4(3*H*)-ones 3c and 3m were similar in terms of inhibition zone to gentamicin, while the remaining analogs exhibited larger inhibition zones compared to the standard. Synthesized motifs 3a–m also showed notable activity against *S. typhimurium*, with ZOIs ranging from 23.00 ± 0.61 mm to 35.00 ± 0.98 mm, while the ZOI of the gentamicin standard was 26.00 ± 0.62 mm. Given that salmonellosis is a major foodborne zoonosis of global public health concern, some of the reported compounds might serve as potential alternatives to gentamicin for the treatment of infections originating from *S. typhimurium* (Campos et al., 2019).

General sensitivity tests were additionally carried out against four fungi, namely, *A. flavus* (LIO), *A. niger* (LIO), *C. albicans* (ATCC 10231), and *R. nigricans* (LIO), with ketoconazole used as the positive control. The results were measured in the form of zones of inhibition, documented in millimeters (Table 5). No resistance was observed in the course of serial screening of quinazolin-4(3*H*)-one 3a–m against all four fungi. The smallest and largest ZOIs against *C. albicans* were found in 3b (30.00 ± 0.91 mm) and 3m (57.00 ± 1.35 mm), respectively, while the ZOI of ketoconazole against *C. albicans* was 57.00 ± 1.36 mm. This finding placed thiophene-containing quinazolin-4(3*H*)-one 3m on a higher pedestal than other members of the series and also revealed that 3m could compare favorably with ketoconazole in the treatment of infection associated with *C. albicans* activity, provided that cytotoxicity reveals tolerability. The antifungal activity of 3a–m against *A. niger* showed ZOI values ranging from 10.00 ± 0.40 mm for 3g to 45.00 ± 1.22 mm for 3h. These findings indicated that 61.5% of the compounds had ZOIs ≥ that of ketoconazole (ZOI = 40.00 ± 1.18) against *A. niger*. Additionally, every member of series 3a–m exhibited growth inhibition against *A. flavus*, with ZOIs ranging from 30.00 ± 0.91 mm for 3c to 42.00 ± 1.12 mm for 3k. Although the ZOI of ketoconazole (47.00 ± 1.28 mm) was larger than those of 3a–m, the antifungal activity of the latter on *A. flavus* was quite encouraging because no ZOI was below 30 mm. Finally, the growth-inhibitory potential of series 3a–m against *R. nigricans* was quite commendable, with ZOIs varying from 25.00 ± 0.68 mm for 3h to 40.00 ± 1.20 mm for 3m, while the ZOI of the ketoconazole standard was 25.00 ± 0.68 mm. This indicated that compound 3h compared favorably with ketoconazole in terms of growth inhibition of *R. nigricans*. It is also worth noting that 85% of the synthesized compounds 3a–m had larger ZOIs than ketoconazole against *R. nigricans*. Among the group, compound 3m emerged as the most active antifungal agent.

**TABLE 5:** Results of *in vitro* antifungal screening, in the form of zones of inhibition (ZOIs; mm), and minimum inhibitory concentration testing on fungal isolates.

S/N	Sample code	*C. albicans*		*A. niger*		*A. flavus*		*R. nigricans*	
		ZOI (mm)	MIC (μg/mL)	ZOI (mm)	MIC (μg/mL)	ZOI (mm)	MIC (μg/mL)	ZOI (mm)	MIC (μg/mL)
1	**3a**	30.00 ± 0.90	62.50	40.00 ± 1.18	15.62	30.00 ± 0.90	31.25	40.00 ± 1.18	15.62
2	**3b**	30.00 ± 0.91	7.81	30.00 ± 0.90	7.81	30.00 ± 0.90	15.62	35.00 ± 1.11	7.81
3	**3c**	30.00 ± 0.90	7.81	35.00 ± 1.11	15.62	30.00 ± 0.91	31.25	35.00 ± 1.10	15.62
4	**3d**	38.00 ± 1.12	7.81	42.00 ± 1.20	15.62	40.00 ± 0.90	31.25	40.00 ± 1.18	7.81
5	**3e**	30.00 ± 0.90	31.25	40.00 ± 1.18	15.62	30.00 ± 0.90	62.50	40.00 ± 1.18	15.62
6	**3f**	35.00 ± 1.12	3.90	40.00 ± 1.18	7.81	40.00 ± 1.20	15.62	40.00 ± 1.18	15.62
7	**3g**	40.00 ± 1.18	3.90	10.00 ± 0.40	15.62	30.00 ± 0.90	15.62	30.00 ± 0.90	31.25
8	**3h**	35.00 ± 1.10	7.81	45.00 ± 1.22	15.62	30.00 ± 0.92	31.25	25.00 ± 0.68	62.50
9	**3i**	35.00 ± 1.10	7.81	35.00 ± 1.11	15.62	40.00 ± 1.20	7.81	35.00 ± 1.10	15.62
10	**3j**	25.00 ± 0.67	15.62	30.00 ± 0.90	7.81	35.00 ± 1.11	15.62	40.00 ± 1.19	7.81
11	**3k**	32.00 ± 0.90	31.25	45.00 ± 1.12	31.25	42.00 ± 1.12	7.81	28.00 ± 0.88	15.62
12	**3L**	30.00 ± 0.90	7.81	40.00 ± 1.18	7.81	35.00 ± 1.10	15.62	40.00 ± 1.18	7.81
13	**3m**	57.00 ± 1.35	3.90	40.00 ± 1.19	3.90	35.00 ± 1.12	7.81	40.00 ± 1.20	3.90
14	**KCZ**	57.00 ± 1.36	3.13	40.00 ± 1.18	3.13	47.00 ± 1.28	6.25	25.00 ± 0.68	6.25

Mean ± SD, standard deviation of triplicate measurements. *C. albicans = Candida albicans* (ATCC, 10231); *A. niger = Aspergillus niger* (LIO); *A. flavus = Aspergillus flavus* (LIO); *R. nigricans = Rhizopus nigricans* (LIO, but sequenced); ATCC, american type culture collection; LIO, locally isolated organism; KCZ, ketoconazole; ZOI, zone of inhibition; MIC, minimum inhibitory concentration.

Owing to the broad spectra of activities observed in general sensitivity testing and the need to identify the lowest growth-inhibitory concentration of the synthesized compounds in relation to the organisms involved, minimum inhibitory concentration (MIC) testing was carried out, utilizing dilution factors to arrive at varying concentrations, namely, 500, 250, 125, 62.50, 31.25, 15.62, 7.81, 3.90, and 1.95 μg/mL. Table 4 shows the results of MIC testing of quinazolin-4(3*H*)-one motifs 3a–m against the four bacterial isolates. The results indicated that the MICs determined for the prepared analogs against *S mutans* varied from 3.90 μg/mL to 62.50 μg/mL, indicating motif 3g to be the most potent against *S. mutans*. This validates the results obtained in the *in silico* study, in which compound 3g possessed a strong binding affinity in the docking model. MIC values for 3a–m against *S. aureus* were between 1.95 μg/mL and 31.25 μg/mL, with compound 3m being the most potent against *S. aureus*. However, the best hits in the *in silico* study (motifs 3c and 3e) also showed excellent MIC values of 3.90 μg/mL. MIC testing against the Gram-negative bacterial strain of *E. coli* elicited MIC values between 3.90 μg/mL and 125.00 μg/mL, indicating motif 3k to be the most potent against *E. coli*. Screening of the motifs against *S. typhimurium* revealed MIC values in the range of 7.81 μg/mL to 125.00 μg/mL, confirming compound 3h to be the most efficacious against *S. typhimurium*. This also validates the results obtained in the *in silico* study, in which compound 3h possessed a strong binding affinity in the 4KR4 docking model. In a similar vein, Table 5 also shows the results of MIC testing of the targeted compounds 3a–m, carried out on four different fungi. MIC results, indicating the action of targeted motifs 3a–m, varied from 3.90 μg/mL to 62.50 μg/mL against *C. albicans* and *R. nigricans*; from 3.90 μg/mL to 31.25 μg/mL against *A. niger*; and 7.18 μg/mL to 62.50 μg/mL against *A. flavus*.

### 3.5 Structure–activity relationship (SAR) study

**FIGURE 6:**
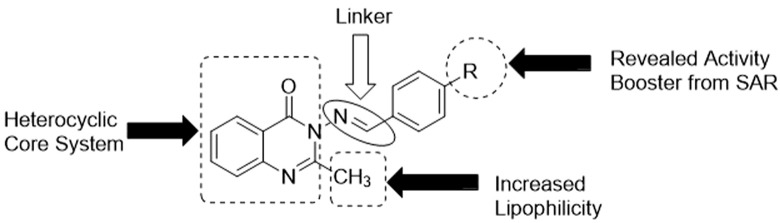
Some essential features required for activity, as shown by the SAR study.

Since the targeted compounds 3a–l were structurally related from their quinazolin-4(3H)-one core nucleus up to the benzylidene side chain, while 3m had its ylide moieties as a thiophenyl template (which is a heterocyclic pharmacophore), it was necessary to establish the effect of the nature of the substituent, as well as its positions of attachment to the aryl nucleus, via a structure–activity relationship (SAR) study (Figure 6). The activity potential of series 3a–m against *S. mutans* varied in the order: 3g > 3h ≈ 3k > 3f ≈ 3j > 3d > 3a ≈ 3c ≈ 3m. The most active compound in this scenario was 3g (4-N(CH_3_)_2_), followed by 3h (4-OH) and 3k (OC_2_H_5_), while 3f (4-Cl) exhibited a drastic reduction in activity against *S. mutans*. This indicates that the presence of electron-donating groups (EDGs) in position 4 of the phenyl group played a paramount role in boosting activity, while in contrast, the presence of an electron-withdrawing group (EWG), such as Cl, in this same position was antagonistic to bioactivity against *S. mutans*. Since a lower MIC implies greater efficacy, the order of the potency trend of the series of compounds 3a–m against *S. aureus* was 3m > 3c ≈ 3e ≈ 3k > 3j > 3a ≈ 3f ≈ 3h ≈ 3l > 3d ≈ 3i. It was observed that compound 3m (MIC = 1.95 μg/mL), with a thiophene moiety, was the most active against *S. aureus*, and there was no consistent pattern for the contribution of either EDG or EWG. This was a clear-cut indication that the replacement of the benzylidene in 3a–l with the heterocyclic thiophene in 3m was a worthwhile advancement in terms of fighting S. aureus-related infections.

The efficacy of the set of synthesized compounds 3a–m against *E. coli* was revealed to vary in the order 3k > 3j > 3d ≈ 3h ≈ 3l ≈ 3m > 3e ≈ 3f ≈ 3i > 3c. Compound 3k, with an EDG (4-OEt), was the most active, with an MIC of 3.90 μg/mL, followed by other members (3j, 3d, 3l, and 3h) also having an EDG in position 4. There was a sharp decrease in the activity of 3c (2-Cl), with an MIC of 125.00 μg/mL, and no activity at all in the case of 3b (2-NO2). This showed that the presence of EWGs (Cl, NO2) on position 2 of the phenyl group resulted in a loss of activity against *E. coli*. Finally, the SAR study showed that the trend of activity against *S. typhimurium* was as follows: 3h > 3k > 3i ≈ 3l ≈ 3m > 3a ≈ 3c ≈ 3d ≈ 3e ≈ 3j > 3f. Once again, motif 3h (MIC = 7.81 μg/mL), bearing an EDG (4-OH), was the most active, followed by another electron-donating agent, 3k (MIC = 15.62 μg/mL), possessing an EDG (4-OEt). The poorest level of activity against *S. typhimurium* was recorded for compound 3f (MIC = 125.00 μg/mL), which clearly had an EWG (4-Cl). Thus, compound 3k emerged as the most potent antibacterial agent across all four bacterial strains.

Regarding the SAR study of antifungal activities, it is interesting to note that compound 3m was the most active antifungal agent against *C. albicans*, *A. niger*, and *R. nigricans* (MIC = 3.90 μg/mL), and also against *A. flavus* (MIC = 7.81 μg/mL). This indicates the importance of heterocyclic thiophene availability in enhancing antifungal activity. It is therefore structurally confirmed that thiophene is a more effective pharmacophoric unit than benzylidene in terms of antifungal activity enhancement.

### 3.6 ADMET studies

Drug attrition is a major challenge that has been attributed to poor pharmacokinetics and toxicity of proposed drug candidates (Agamah et al., 2020). The ADMETLAB tool was employed to predict the drug-likeness and medicinal chemistry-related properties of the substituted arylidene-based quinazolin-4(3*H*)-one motifs 3a–m (Table 6). Compound 3m possessed the highest QED score of 0.795, which is a measure of drug-likeness. Based on synthetic accessibility, it was observed that all the compounds had SA scores lower than 6, further confirming their ease of synthesis, which makes reproducibility possible. All the compounds had parameters within the acceptable range (MW ≤ 500; logP≤5; HBA≤10; HBD≤5) for the Lipinski rule, suggesting that the compounds had properties that qualified them as possible drug candidates. According to the Pfizer rule, compounds with low TPSA (<75) and high log P (>3) are considered excellent drug candidates. On these criteria, only compounds 3b, 3e, 3h, 3l, and 3m had acceptable values. However, all the compounds exhibited acceptable values according to the GSK rule (logP ≤4; MW ≤ 400). All the substituted quinazolin-4(3*H*)-one motifs exhibited high gastrointestinal absorption, suggesting their ability to be easily absorbed in the gastrointestinal tract and thus their oral availability. Overall, all the synthesized compounds have relatively good predicted pharmacodynamics and pharmacokinetic properties, making them strong candidates as potential antimicrobial drugs.

**TABLE 6:** Predicted drug-likeness, medicinal chemistry, and pharmacokinetic properties of the substituted quinazolin-4(3H)-one motifs, determined using ADMETLAB.

Sample code	RB	HBA	HBD	MR	TPSA	c Log P	QED	SA	Lipinski rule	Pfizer rule	GSK rule	GIA	BBB permeant	Pgp substrate
**3a**	2	3	0	80.4	47.25	2.91	0.667	1.970	ACPT	RJCT	ACPT	High	Yes	No
**3b**	3	5	0	89.22	93.07	2.18	0.422	2.227	ACPT	ACPT	ACPT	High	No	No
**3c**	2	3	0	85.41	47.25	3.45	0.682	2.097	ACPT	RJCT	ACPT	High	Yes	No
**3d**	3	4	0	86.89	56.48	2.93	0.697	2.063	ACPT	RJCT	ACPT	High	Yes	No
**3e**	2	4	1	82.43	67.48	2.53	0.732	2.167	ACPT	ACPT	ACPT	High	Yes	No
**3f**	2	3	0	85.41	47.25	3.45	0.682	2.032	ACPT	RJCT	ACPT	High	Yes	No
**3g**	3	3	0	94.61	50.49	2.94	0.699	2.170	ACPT	RJCT	ACPT	High	Yes	No
**3h**	2	4	1	82.43	67.48	2.51	0.732	2.115	ACPT	ACPT	ACPT	High	Yes	No
**3i**	3	3	0	90.18	47.25	3.57	0.696	2.058	ACPT	RJCT	ACPT	High	Yes	No
**3j**	3	4	0	86.89	56.48	2.93	0.697	2.011	ACPT	RJCT	ACPT	High	Yes	No
**3k**	4	4	0	91.7	56.48	3.24	0.696	2.054	ACPT	RJCT	ACPT	High	Yes	No
**3l**	3	5	1	88.92	76.71	2.5	0.753	2.181	ACPT	ACPT	ACPT	High	Yes	No
**3m**	3	3	0	77.45	72.55	2.62	0.795	2.853	ACPT	ACPT	ACPT	High	Yes	No

RB: rotatable bond; HBA: hydrogen bond acceptor; HBD: hydrogen bond donor; MR: molar refractivity; TPSA: topological polar surface area; ACPT: accept; RJCT: reject; QED: measure of drug-likeness; SA: synthetic accessibility score; GIA: gastro-intestinal absorption; BBB: blood–brain barrier; Pgp substrate: P-glycoprotein.

## 4 Conclusion

All the members of the array of synthesized targeted quinazolin-4(3*H*)-one analogs 3a–m were successfully synthesized with good to excellent yields. The chemical structures were validated using physicochemical parameters and spectroscopic characterization. *In silico* testing of antimicrobial potential revealed that the compounds had good binding affinities, and in some cases, even better than those of the standards gentamicin and ketoconazole, which were used as references. The results of the *in silico* studies were further validated via *in vitro* antimicrobial screening, and it was observed that some of the best hits from the molecular docking studies displayed good MIC values. The antimicrobial potential of the synthesized analogs was investigated through *in vitro* screening with standard procedures. 2-methyl-3-((thiophen-2-ylmethylene)amino)quinazolin-4(3*H*)-one 3m emerged as the most active antibacterial agent (with an MIC value of 1.95 μg/mL) against *S. aureus*, while the same compound 3m was also the most active antifungal agent, with an MIC value of 3.90 μg/mL against *C. albicans*, *A. niger*, and *R. nigricans*. Hence, this work has identified candidates for further study with the aim of investigating the pharmacokinetic–pharmacodynamic profiles of these analogs for possible future drug discovery.

## Data Availability

The original contributions presented in the study are included in the article/Supplementary Material, further inquiries can be directed to the corresponding author.
