# Supplementary material for: Synthesis, in silico and in vitro antimicrobial efficacy of substituted arylidene-based quinazolin-4(3H)-one motifs
Source: Front Chem. 2023 Sep 25;11:1264824. doi: 10.3389/fchem.2023.1264824 (PMC10561392; doi:10.3389/fchem.2023.1264824)
Supplement: Supplementary file 1 [file DataSheet1.docx]

Supplementary Material

1. **1H-NMR and ^13^C-NMR spectra of some of the synthesized compounds**


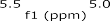

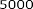

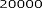

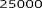

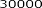


**Supplementary Figure 1**: 1H-NMR Spectrum of Compound **1** in DMSO-d_6_ at 500 MHz


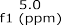

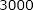

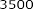


**Supplementary Figure 2**: 1H-NMR Spectrum of Compound **2** in DMSO-d_6_ at 500 MHz


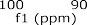

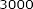

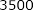

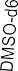


**Supplementary Figure 3**: 13C-NMR Spectrum of Compound **2** in DMSO-d_6_ at 125 MHz


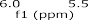

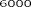

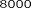

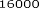

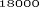

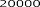

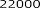

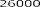

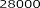

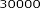

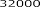

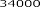

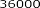

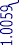

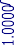

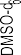


**Supplementary Figure 4**: 1H-NMR Spectrum of Compound **3a** in DMSO-d_6_ at 400 MHz


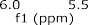

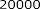

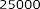

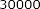

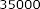

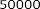

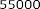


**Supplementary Figure 5**: 1H-NMR Spectrum of Compound **3g** in DMSO-d_6_ at 400 MHz


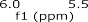

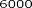

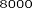

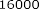

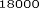

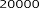

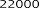

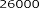

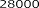

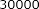

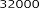

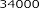

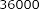

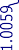

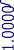


**Supplementary Figure 6**: 1H-NMR Spectrum of Compound **3h** in DMSO-d_6_ at 400 MHz


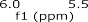

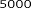

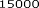

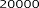

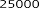

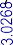


**Supplementary Figure 7**: 1H-NMR Spectrum of Compound **3j** in DMSO-d_6_ at 400 MHz


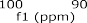

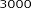

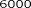

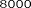

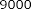

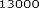

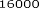

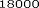

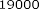

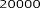


**Supplementary Figure 8**: 13C-NMR Spectrum of Compound **3j** in DMSO-d_6_ at 400 MHz

**Supplementary Figure 9**: 1H-NMR Spectrum of Compound **3m** in DMSO-d_6_ at 400 MHz


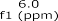

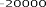

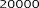

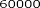

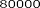

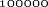

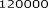

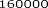

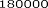

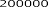

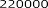

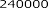

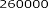

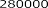


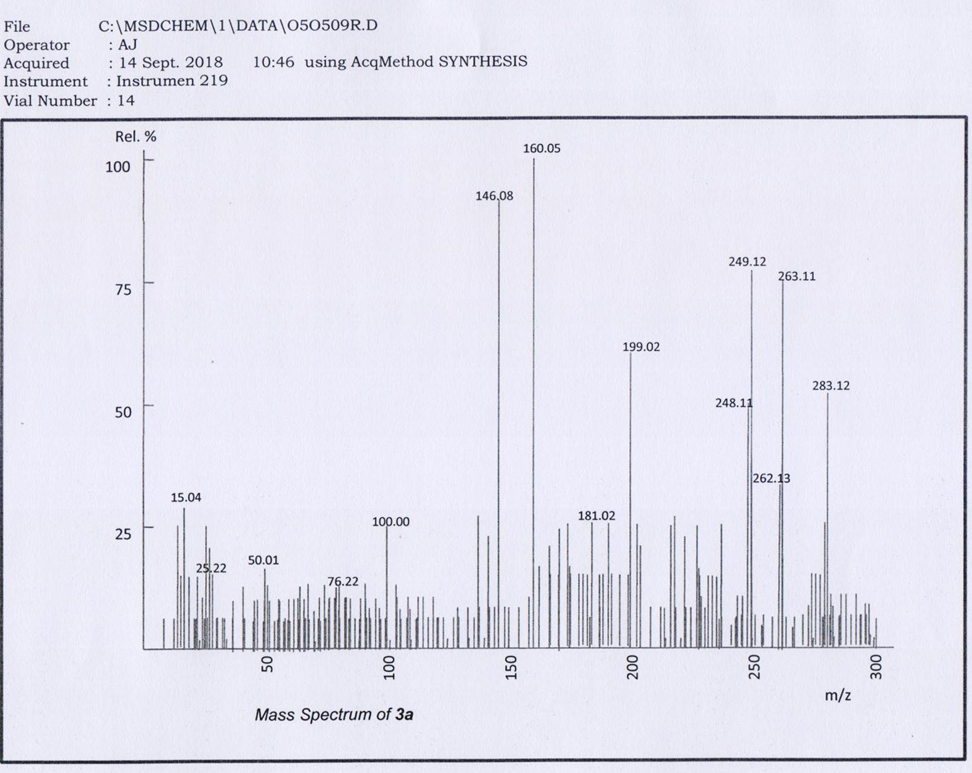


**Supplementary Figure 10**: Mass Spectrum of Compound **3a**.

**2.0 *In vitro* Antimicrobial Activity**

**2.1 Antibacterial sensitivity testing of compounds,** **3a-m**

All the synthesized compounds **(3a-m)** and Gentamicin were screened for antibacterial activity on two gram positive (*Streptococcus mutans* and *Staphylococcus aureus*) and two gram negative (*Escherichia coli* and *Salmonella typhimurium*) bacterial strains using agar well diffusion method. The medium employed was diagnostic sensitivity test agar (Biotech Ltd). With the aid of a sterile 1 ml pipette, about 0.2 ml of the broth culture of test organism was added to 18 ml sterile molten diagnostic sensitivity test agar (Biotech Ltd) which had already cooled down to 45 °C. This was well mixed and poured into previously sterilized petri dishes, which had been properly labeled according to the test organisms. The medium was then allowed to set. With the aid of a sterile cork borer, the required numbers of holes were bored into the medium. The wells were made of about 5 mm to the edge of the plate. The wells were then filled up aseptically with the solution of the compound in DMSO using Pasteur pipettes. Gentamicin was used as the standard antibacterial agent at a concentration of 1000 µg/ml. The plates were allowed to stand for about 1 h on the bench for proper diffusion of the antibacterial agents into the medium and then incubated uprightly at 37 °C for 24 h. Care was taken not to stockpile the plates. Clear zones of inhibition in millimeters indicated the relative susceptibility of the bacteria to the compounds **(3a-m)** and Gentamicin standard.

**2.2 Determination of minimum inhibitory concentration (MIC)**

The Minimum Inhibitory Concentration (MIC) was done using the method of Russell and Furr. Based on the level of resistance of some organisms and large zones of inhibition experienced in others, Minimum Inhibitory Concentration (MIC) was selectively done for the bacterial strains. Different concentrations (between 3.90 µg/ml and 1000.0 µg/ml) of the compounds and standard were prepared using a twofold dilution which was prepared in a sterile plate with the aid of sterile pipette and then mixed with 18 ml of molten nutrient agar. This was then allowed to set. The surface of the nutrient agar plate was allowed to dry before streaking with overnight broth cultures of the bacterial strains. The plates were then labeled accordingly and incubated at 37 °C for up to 72 h. They were subsequently examined for the presence or absence of growth. The lowest concentration preventing the growth of bacteria was taken as the Minimum Inhibitory Concentration of the compounds. This procedure was likewise repeated for the Gentamicin (standard).

**2.3 Determination of antifungal activity**

The antifungal activities of the synthesized compounds were determined using four organisms: *C. albicans*, *A. niger*, *A. flavus*, *R. nigricans*. These were determined by placing one disc (3 mm diameter) of a 3 day old culture of the organisms in each of the triplicates petri dishes (11 cm diameter) with 160 ml Potato Dextrose Agar (PDA) medium and 3 ml of the different synthesized compounds **(3a-m)**. The control experiments were set up with 3 ml of ketoconazole using same media. This was done on triplicate plates and was incubated at room temperatures (28 ± 2 °C) for three days. Daily measurements of the mycelia extension of the cultures were determined by measuring culture along diameters and comparing with mycelial growth of the control. The difference in their diameters reflects the extent of inhibition by the synthesized compounds.

1. **Molecular Dynamics Simulation**

**Table S1 RMSD analyses for the protein-ligand complexes (Predicted antibacterial activities)**

| **S/N** | **Protein-ligand complex** | **Average** | **SD** | **Min** | **Max** |
| --- | --- | --- | --- | --- | --- |
| 1. | 4TQX in the 4TQX-3g complex | 3.903 | 0.903 | 0.065 | 5.386 |
|  | 3g in the 4TQX-3g complex | 1.193 | 0.527 | 0.081 | 2.648 |
| 2. | 2XCS in the 2XCS-3c complex | 2.421 | 0.633 | 0.048 | 3.506 |
|  | 3c in the 2XCS-3c complex | 3.595 | 0.623 | 0.155 | 5.477 |
| 3. | 2XCS in the 2XCS-3e complex | 2.471 | 0.422 | 0.080 | 3.319 |
|  | 3e in the 2XCS-3c complex | 2.775 | 0.524 | 0.163 | 4.385 |
| 4. | 2VF5 in the 2VF5-3k complex | 1.386 | 0.130 | 0.184 | 1.752 |
|  | 3k in the 2VF5-3k complex | 0.895 | 0.191 | 0.055 | 1.615 |
| 5. | 2VF5 in the 2VF5-3l complex | 1.471 | 0.125 | 0.176 | 1.721 |
|  | 3l in the 2VF5-3l complex | 1.570 | 0.284 | 0.092 | 2.921 |
| 6. | 4KR4 in the 4KR4-3i complex | 2.284 | 0.149 | 0.050 | 2.532 |
|  | 3i in the 4KR4-3i complex | 4.737 | 1.637 | 0.028 | 7.594 |
| 7. | 4KR4 in the 4KR4-3h complex | 2.300 | 0.174 | 0.050 | 2.533 |
|  | 3h in the 4KR4-3i complex | 2.579 | 0.474 | 0.023 | 5.278 |

**Table S2 RMSD analyses for the protein-ligand complexes (Predicted antifungal activities)**

| **S/N** | **Protein-ligand complex** | **Average** | **SD** | **Min** | **Max** |
| --- | --- | --- | --- | --- | --- |
| 1. | 1IYL in the 1IYL -3c complex | 1.950 | 0.227 | 0.087 | 2.347 |
|  | 3c in the 1IYL -3c complex | 0.788 | 0.320 | 0.082 | 1.991 |
| 2. | 3K4Q in the 3K4Q -3h complex | 1.514 | 0.166 | 0.081 | 1.824 |
|  | 3h in the 3K4Q -3h complex | 3.972 | 0.460 | 0.124 | 5.563 |
| 3. | 3K4Q in the 3K4Q -3m complex | 1.404 | 0.099 | 0.080 | 1.636 |
|  | 3h in the 3K4Q -3m complex | 4.304 | 0.755 | 0.163 | 6.084 |
| 4. | 4YNT in the 4YNT-3b complex | 2.165 | 0.499 | 0.071 | 3.234 |
|  | 3b in the 4YNT-3b complex | 2.798 | 0.727 | 0.123 | 4.744 |
| 5. | 4YNT in the 4YNT-3i complex | 1.362 | 0.101 | 0.070 | 1.522 |
|  | 3i in the 4YNT-3i complex | 1.639 | 0.263 | 0.041 | 2.253 |
| 6. | 4YNT in the 4YNT-3k complex | 1.369 | 0.111 | 0.071 | 1.567 |
|  | 3k in the 4YNT-3k complex | 0.906 | 0.227 | 0.094 | 1.936 |
| 7. | 4YNT in the 4YNT-3m complex | 1.544 | 0.147 | 0.070 | 1.746 |
|  | 3m in the 4YNT-3m complex | 1.543 | 0.216 | 0.070 | 2.697 |
| 8. | AFA0A367KUY9 in the AFA0A367KUY9-3b complex | 1.473 | 0.157 | 0.064 | 1.708 |
|  | 3b in the AFA0A367KUY9-3b complex | 1.116 | 0.183 | 0.082 | 1.806 |
